# Arenavirus Glycan Shield Promotes Neutralizing Antibody Evasion and Protracted Infection

**DOI:** 10.1371/journal.ppat.1005276

**Published:** 2015-11-20

**Authors:** Rami Sommerstein, Lukas Flatz, Melissa M. Remy, Pauline Malinge, Giovanni Magistrelli, Nicolas Fischer, Mehmet Sahin, Andreas Bergthaler, Sebastien Igonet, Jan ter Meulen, Dorothée Rigo, Paolo Meda, Nadia Rabah, Bruno Coutard, Thomas A. Bowden, Paul-Henri Lambert, Claire-Anne Siegrist, Daniel D. Pinschewer

**Affiliations:** 1 Department of Pathology and Immunology, University of Geneva, Geneva, Switzerland; 2 World Health Organization Collaborating Centre for Vaccine Immunology, University of Geneva, Geneva, Switzerland; 3 Division of Experimental Virology, Department of Biomedicine, University of Basel, Basel, Switzerland; 4 Novimmune SA, Plan-Les-Ouates, Switzerland; 5 Institut Pasteur, Département de Virologie, Unité de Virologie Structurale and CNRS UMR 3569 Virologie, Paris, France; 6 Institute of Virology, Philipps University Marburg, Marburg, Germany; 7 Department of Cell Physiology and Metabolism, University of Geneva, Geneva, Switzerland; 8 AFMB, UMR7257 CNRS/Aix Marseille Université, Marseille, France; 9 Division of Structural Biology, Wellcome Trust Centre for Human Genetics, University of Oxford, Oxford, United Kingdom; National Institutes of Health, UNITED STATES

## Abstract

Arenaviruses such as Lassa virus (LASV) can cause severe hemorrhagic fever in humans. As a major impediment to vaccine development, delayed and weak neutralizing antibody (nAb) responses represent a unifying characteristic of both natural infection and all vaccine candidates tested to date. To investigate the mechanisms underlying arenavirus nAb evasion we engineered several arenavirus envelope-chimeric viruses and glycan-deficient variants thereof. We performed neutralization tests with sera from experimentally infected mice and from LASV-convalescent human patients. NAb response kinetics in mice correlated inversely with the N-linked glycan density in the arenavirus envelope protein’s globular head. Additionally and most intriguingly, infection with fully glycosylated viruses elicited antibodies, which neutralized predominantly their glycan-deficient variants, both in mice and humans. Binding studies with monoclonal antibodies indicated that envelope glycans reduced nAb on-rate, occupancy and thereby counteracted virus neutralization. In infected mice, the envelope glycan shield promoted protracted viral infection by preventing its timely elimination by the ensuing antibody response. Thus, arenavirus envelope glycosylation impairs the protective efficacy rather than the induction of nAbs, and thereby prevents efficient antibody-mediated virus control. This immune evasion mechanism imposes limitations on antibody-based vaccination and convalescent serum therapy.

## Author Summary

Neutralizing antibodies (nAbs) represent a key principle of antiviral immunity. Protective vaccines aim at inducing nAbs to prevent viral infection, and infusion of nAbs in convalescent patient serum can offer a potent antiviral therapy. Certain viruses, however, have found ways to evade nAb control. Amongst them are high-risk pathogens of the arenavirus family such as Lassa virus (LASV), which is a frequent cause of hemorrhagic fever in West Africa. Here we unveil the molecular strategy by which arenaviruses escape antibody neutralization and avoid efficient immune control. We show that their surface is decorated with sugar moieties, serving to shield the virus against the neutralizing effect of the host’s antibodies. This immune evasion strategy differs from those described for other viruses, in which sugars impair primarily the induction of antibodies or allow for viral mutational escape. The arenavirus sugar coat renders the host nAb response inefficient and as a consequence thereof, the host fails to promptly control the infection. Our results offer a compelling explanation for the long history of failures in trying to make a nAb-based vaccine against LASV or in using convalescent serum for therapy. These mechanistic insights will support vaccine development efforts against arenaviruses such as LASV.

## Introduction

For most viral vaccines in clinical use today, neutralizing antibodies (nAbs) represent the main correlate of protection [1, 2]. However, viral immune evasion strategies such as antigenic variation and so-called “glycan shields” on viral envelope proteins [3–8] can undermine the protective, neutralizing capacity of antibody immunity. An understanding of the mechanisms underlying viral interference with the host’s antibody defense is, therefore, of pivotal importance to refine vaccination strategies.

Members of the *Arenaviridae* are found worldwide, reflecting the geographic distribution of each virus’ natural rodent host [9]. Several arenaviruses, categorized as high-risk pathogens, can cause lethal hemorrhagic fever in humans and require biosafety level 4 containment. Most prominently, Lassa virus (LASV) is endemic in West Africa and accounts for estimated 300’000 human infections with several thousand deaths each year [10]. Similarly, the South American clade B viruses Junin (JUNV), Guanarito, Machupo and Sabia virus cause Argentine, Venezuelan, Bolivian and Brazilian hemorrhagic fever, respectively. Despite these viruses’ socio-economic impact, the live-attenuated JUNV strain Candid #1 [11] remains the only arenavirus vaccine in clinical use [12]. Besides life-supporting intensive care, ribavirin is the only therapeutic option in Lassa fever but shows limited efficacy [13]. Hence the development of a LASV vaccine remains a priority.

The human B cell response to LASV infection allows for a timely diagnosis by immunofluorescence and complement fixation [14]. But the kinetics of such non-protective, binding antibody responses contrast with those of nAbs. Already shortly after the identification of Lassa virus in the early 1970ies, Casals and colleagues noted a “lack of synchrony in the development of antibodies detected by the different tests” [14]. Indeed, nAbs are undetectable in the first two to three months after the onset of clinical symptoms, and in most patients remain at or below the 1:100 titer range throughout several months of follow-up [15]. With most convalescent serum donors never reaching an effective titer range [15, 16], passive serum therapy in human LASV infection evidenced only limited efficacy [17]. Intriguingly, the discrepancy between binding and neutralizing antibodies was also observed in monkeys immunized with gamma-irradiated Lassa virions [18]. This argued against infection-associated lymphoid depletion and immunosuppression as sole reasons for poor LASV nAb induction [19, 20]. In contrast to LASV, passive serum therapy represents an efficient treatment against Argentine hemorrhagic fever [21] and formalin-inactivated JUNV, unlike LASV, can induce potent nAb responses [22]. The reasons underlying differential behavior of JUNV and LASV have remained unclear though.

A serological response pattern analogous to the one of humans to LASV is observed when mice are infected with lymphocytic choriomeningitis virus (LCMV), a close relative of LASV. Antibodies binding to the nucleoprotein (NP) and the glycoprotein-2 subunit (GP-2) are elicited early after infection and reach high titers, whereas neutralizing antibodies target exclusively GP-1 [23] and remain undetectable for the first 40–60 days after infection [24–26]. Furthermore, nAbs only arise in animals with protracted viremia, which is thought to drive continuous somatic hypermutation and antibody evolution [25]. Using reverse genetic techniques to swap glycoproteins between LCMV and vesicular stomatitis virus, we have previously demonstrated that delayed and weak LCMV-neutralizing antibody induction represents a GP-intrinsic feature [27]. Irrespective of the isolation of rare clones of neutralizing monoclonal antibodies (mAbs) against LCMV [28, 29] and LASV [30] which can exhibit therapeutic efficacy *in vivo* [29], these observations supported the notion of a neutralization evasion mechanism in these Old World arenavirus glycoproteins.

The arenavirus envelope carries a single glycoprotein (GP) complex. It is synthesized as GP-C precursor, which is post-translationally cleaved into a stable signal peptide, an outer globular domain (GP-1) and the membrane-anchored GP-2 stalk. These resulting GP complexes consisting of GP-1, GP-2 and the stable signal peptide remain non-covalently associated on the virion surface and are responsible for receptor binding and membrane fusion. The GPs of LCMV and LASV contain six and seven N-linked glycosylation motifs in GP-1, respectively, all of which are used during protein biosynthesis [31, 32]. This was established in earlier mutagenesis studies using rLCMV [32] and plasmid-based expression of LASV-GP [31], respectively, demonstrating that mutation of each individual N-linked glycosylation site resulted in the predicted reduction in the GP’s molecular mass. Table 1 provides a comparative overview on N-linked glycosylation motifs in thirty arenavirus GP-1 sequences of all clades, which we aligned based on amino acid sequence homology (see also S1 Fig). We numbered the glycans from 1 to 15 (Glc1 –Glc15), to allow for a comparison of homologous glycans in diverse arenaviruses. N-linked glycosylation impacts protein expression and function [33], and thus influences LCMV-GP processing, transport and cell fusion [32]. As an additional potential role, early monoclonal antibody (mAb) work suggested that Glc12 in GP-1 masked a neutralizing epitope [34]. In support of this hypothesis, a recent mutagenesis study with LCMV found that most GP-1 glycans but not Glc9 and Glc12 affected viral fitness [35].

**Table 1:** N-linked glycosylation motifs in arenavirus GP-1 sequences.

Evolutionary Lineage^a^	GP clade	Arenavirus	Acronym	Genbank^b^	Glycans on GP-1^c^	Position of predicted glycans (Glc)^d^														
						1	2	3	4	5	6	7	8	9	10	11	12	13	14	15
OW	-	Lujo	LUJV	FJ952384	6		73			93	104			112		148				194
		Dandenong	DANV	EU136038	7		80	85	95		114			124			171			232
		Lymphocytic choriomeningitis	LCMV	AJ297484 (WE)AY847350 (ARM)	6			85	95		114			124			171			232
		Lassa	LASV	J04324	7			79	89	99	109			119			167			224
		Mobala	MOBV	AY342390	7			78	88	98	108			118			166			224
		Mopeia	MOPV	AY772170	7			78	88	98	108			118			166			222
		Morogoro	MORV	EU914103	7			78	88	98	108			118			166			222
		Ippy	IPPYV	DQ328877	10	66		78	88	97	107			117		159	165	197		228
NW	A	Flexal	FLEV	AF512831	8		74		89		111	116		130			179		223	240
		Allpahuayo	ALLV	AY012687	8		74		89		111		119	130			179		223	240
		Parana	PARV	AF485261	10		74		89		111	116	119	130			179	215	219	240
		Pirital	PIRV	AF277659	9		75		90	101	112	117	122	133			182	218		
		Pichinde	PICV	K02734	11	67	74		89	100	111	116	121	132			181	217		241
NW	C	Oliveros	OLVV	U34248	9		75		90	101	112	117		131			180		234	251
		Latino	LATV	AF485259	10		75		90	101	112	117		131	138		180		231	248
NW	B	Junin Cand#1	JUNV-vacc	HQ126699	3				95	105							178			
		Junin XJ13	JUNV	AY358023	4				95	105						166	178			
		Tacaribe	TCRV	KP159416	4			83	95							164	176			
		Machupo	MACV	AY619643	5			83	95						137	166	178			
		Amapari	AMAV	AF512834	5				88	99				128			174			214
		Guanarito	GTOV	AF485258	5				88					125			174		202	214
		Cupixi	CPXV	AF512832	5				88	99				125			174			214
		Sabia	SABV	U41071	7		69		88	99				125		171	178			222
		Chapare	CHPV	EU260463	7		69		88	99				125		171	178			218
NW-rec	B	Bear Canyon	BCNV	AY924391	5		73		88					130			179			216
		Catarina	CATV	DQ865244	5		73		88					129			180			217
		North American Arena	NAAV	EU123329	5		73		88							168	180			219
		Skinner Tank	SKTV	EU123328	6		73		88					129		168	178			214
		Tamiami	TAMV	AF512828	6		73		88				117	128			179			218
		Whitewater arroyo	WWAV	AF228063	6		73		88					126		165	176			215

^a^ Evolutionary lineage is indicated as OW (Old World) or NW (New World) [9]. rec: recombinant.

^b^ Genbank accession numbers of the virus strains used for GP-1 sequence analysis.

^c^ Predicted N-linked glycans (Glc) were determined according to NX[S/T] motifs (with exclusion of NP[S/T] patterns if any [36]) and numbered 1 to 15 following the alignment of GP-1 sequences of all viruses listed.

^d^ For each N-linked glycosylation motif NX[S/T] the N position is indicated as amino-acid number in the respective GP. Full-length GP-1 amino acid sequence alignments are displayed in S1 Fig.

Here we performed infection experiments with recombinant LCM viruses expressing a range of arenavirus GPs and glycosylation variants thereof. We assessed nAb induction and measured viral sensitivity to neutralization by human and mouse antisera as well as by mAbs. Our findings establish specific viral GP-1 glycans as key mediators of arenavirus nAb evasion in mice and humans. GP-specific antibody responses were readily elicited but reacted predominantly if not exclusively with glycan-deficient viral variants. These observations delineate a viral immune evasion strategy, which prolongs viremia in primary infection and remains to be overcome in antibody-based vaccination against human-pathogenic arenaviruses such as LASV.

## Results

### nAb response kinetics against Clade B arenavirus envelopes correlate with GP-1 glycan density

**Fig 1:**
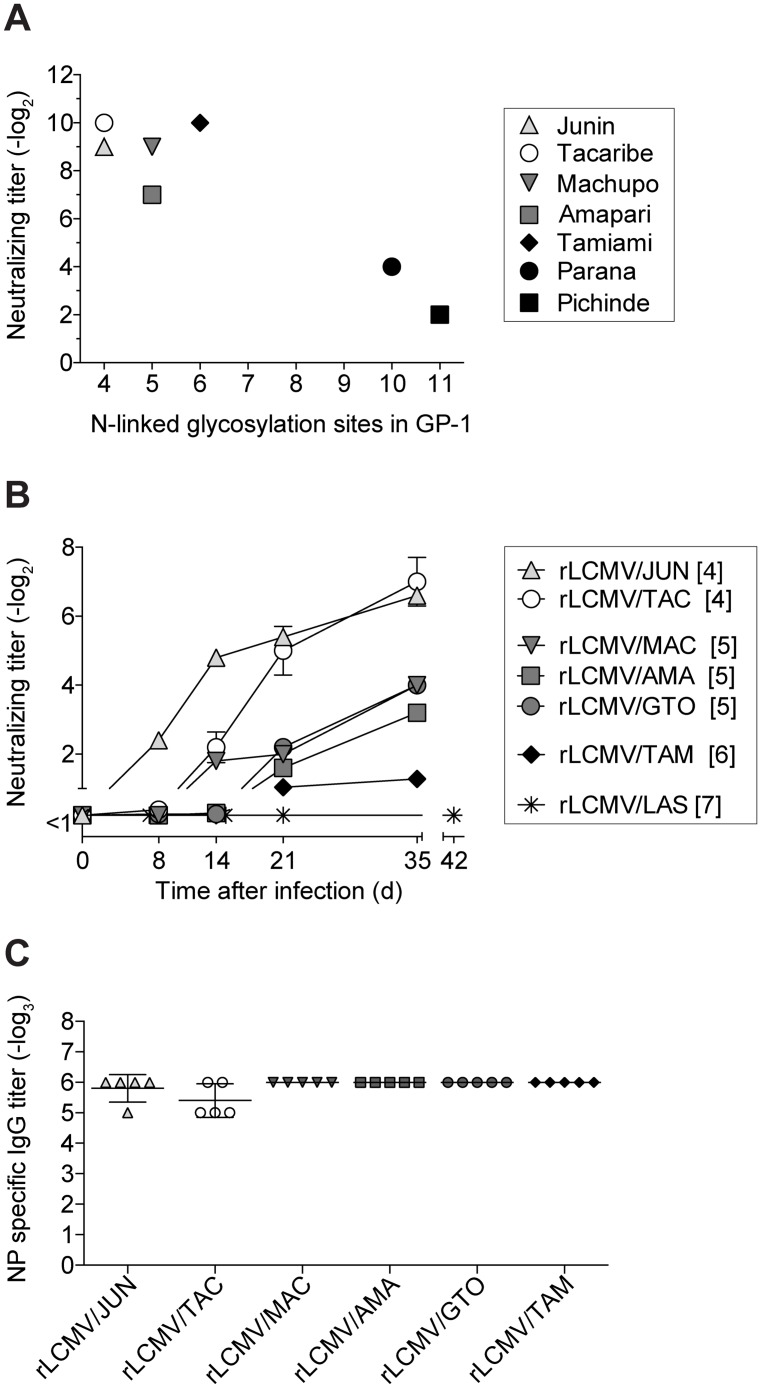
nAb response kinetics against Clade B arenavirus envelopes correlate with GP-1 glycan density. (A) Neutralization titer data published by Trapido *et al*. in 1971 [37] are plotted in relation to the number of N-linked glycosylation motifs in the respective virus’ GP-1 (according to Genbank accession numbers as listed in Table 1). Trapido *et al*. had tested the antiviral neutralizing potency of hamster serum obtained upon hyperimmunization with the respective viruses. (B-C) We infected C57BL/6 mice i.v. with 2x10^5^ PFU of the indicated recombinant LCM viruses carrying a range of clade B arenavirus GPs. rLCMV/LAS data originate from a separate experiment and are included for comparison. The number of GP-1 N-linked glycosylation motifs is indicated in brackets. (B) Serum samples were tested in neutralization assays against the respective virus used for infection. Symbols represent the mean ± SEM of five mice per group. One of two similar experiments is shown. (A-B) Neutralizing titers were determined in 8-fold (A) or 10-fold (B) pre-diluted serum. (C) LCMV-NP specific IgG titers were determined in 100-fold pre-diluted serum on d35. Individual data points and the mean ± SEM of five mice per group are shown.

We sought support for our hypothesis that N-linked glycosylation represented an arenaviral strategy for nAb evasion. A review of historical data documented that nAb induction differed considerably between individual arenaviruses [37]. Interestingly, we noticed that the Pichinde and Parana viruses with 11 and 10 GP-1 glycosylation motifs, respectively, [38] were reported to elicit lower nAb titers than their relatives Tacaribe, Junin, Amapari, Machupo and Tamiami with only 4–6 such motifs (Fig 1A). Not only the GP-1 as molecular target of nAbs, but also the viral backbone could have influenced nAb induction in the infected host. We therefore engineered recombinant LCM viruses (rLCMV), which expressed the Tacaribe, Junin, Amapari, Machupo, Guanarito or Tamiami GPs instead of LCMV-GP. These GPs were chosen because they all were of clade B phylogeny but spanned a range of between four to six predicted N-linked GP-1 glycans. Upon infection of mice with rLCMV carrying either the Tacaribe or Junin GP (rLCMV/TAC, rLCMV/JUN; 4 GP-1 glycans) nAbs were detectable within 8 to 14 days after infection and reached appreciable titers (Fig 1B). rLCMV expressing either the Amapari, Machupo or Guanarito virus GPs (rLCMV/AMA, rLCMV/MACV, rLCMV/GTO; 5 GP-1 glycans) induced detectable nAb responses within 14 to 25 days, with lower titers than elicited against the former two recombinant viruses carrying only 4 GP-1 glycans. Finally, nAbs to rLCMV/TAM (Tamiami virus GP; 6 GP-1 glycans) remained only marginally above technical backgrounds throughout the 35 days observation period. This suggested an inverse correlation between neutralizing antibody responses and the number of GP-1 glycans. Conversely, all Clade B GP-recombinant LCM viruses elicited comparable LCMV-NP-specific antibody titers (Fig 1C), supporting the concept that differential nAb induction was an intrinsic feature of the individual Clade B GPs (Fig 1C). For further comparison to the Clade B GP-recombinant viruses spanning a range of between 4–6 GP-1 glycans, Fig 1B shows also that rLCMV expressing the Old World LASV GP (rLCMV/LAS) with seven GP-1 glycans [38] did not induce any detectable nAbs response within the time frame of our experiment. Altogether, these findings supported the hypothesis that GP-1 glycans represent an impediment to rapid and potent nAb formation by the arenavirus-infected host.

### Viral variants lacking select GP-1 glycans elicit a potent but largely variant-specific nAb response

**Fig 2:**
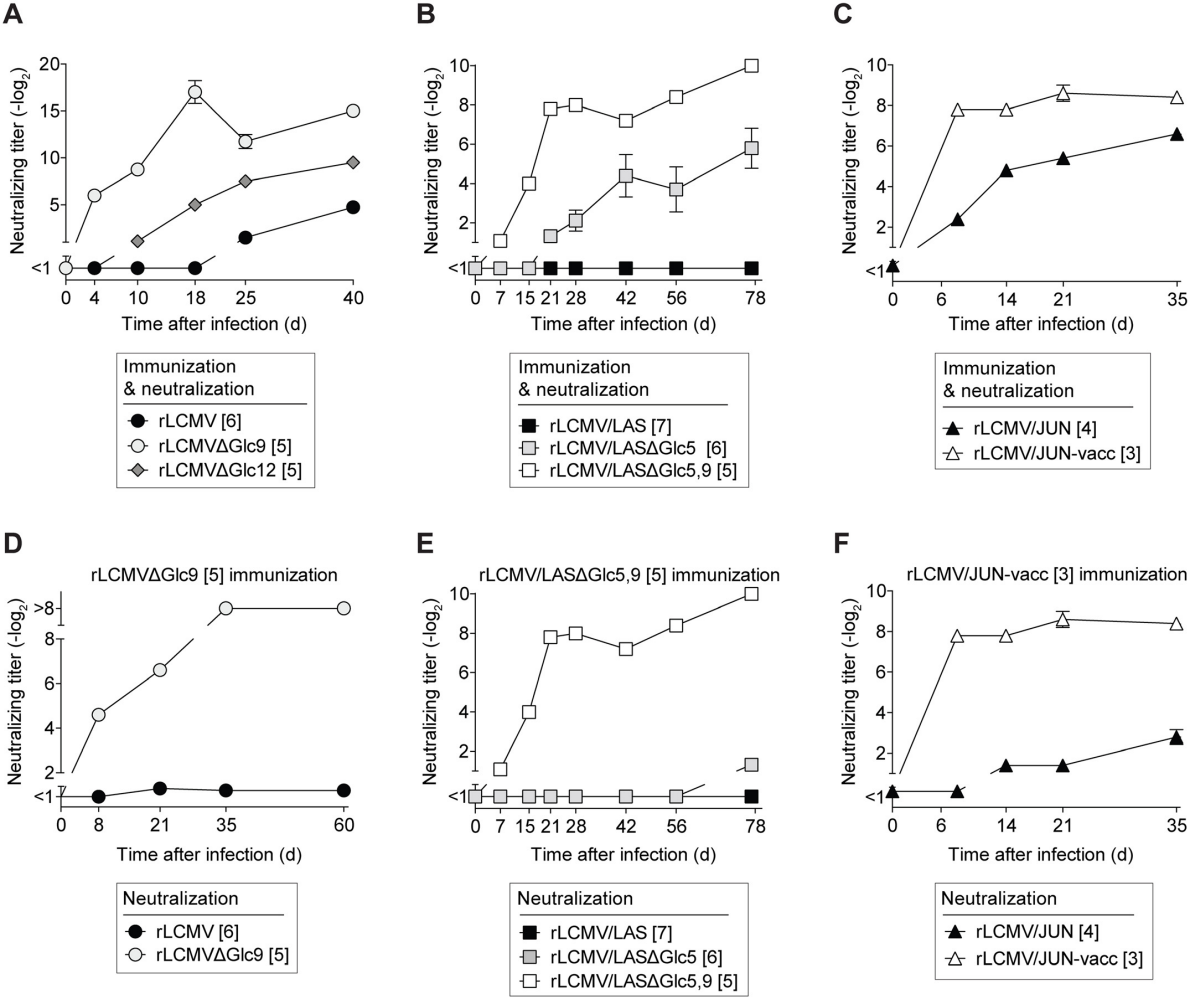
Viral variants lacking select GP-1 glycans elicit a potent but largely variant-specific nAb response. We infected C57BL/6 mice with the indicated viruses and variants expressing partially glycan-deficient GP versions and determined neutralizing serum activity against the immunizing virus (A-C) or against heterologous virus in comparison to the immunizing virus (D-F), as indicated. Doses of 4x10^6^ PFU (A), 5x10^5^ PFU (B, E) or 2x10^5^ PFU (C, D, F) were given as a single i.v. injection on day 0. For each virus, the number of GP-1 N-linked glycosylation motifs on GP-1 of each virus’ GP is indicated in brackets. Symbols represent the mean ± SEM of four to five mice per group. One of two similar experiments is shown. Neutralizing titers were determined in 12.5-fold (A), 10-fold (B-C, E-F) or 8-fold (D) pre-diluted serum.

Glc9- and Glc12-deficient LCMV-GPs reportedly exhibit normal cell surface expression, and the corresponding viruses (rLCMVΔGlc9, rLCMVΔGlc12) grow normally in cell culture [35]. Here we infected mice with rLCMVΔGlc9 or rLCMVΔGlc12 to analyze nAb responses. Both, rLCMVΔGlc9 and rLCMVΔGlc12 induced a more rapid nAb response of higher titer than a cDNA-derived control virus with wt GP (rLCMV, Fig 2A). The effect of Glc9 was, however, more pronounced than the one of Glc12 and we therefore centered the remainder study around Glc9. rLCMVΔGlc9 elicited lower NP-specific antibody responses than rLCMV wt (S2A Fig). This was apparently due to accelerated elimination of rLCMVΔGlc9 and reduced antigen loads over time (see below). Thus, Glc9 deficiency exerted a distinct effect on nAb titers without augmenting antibody responses to the viral backbone. Extending the mutagenesis study to LASV-GP we considered that in addition to the glycans in LCMV-GP, LASV-GP contained Glc5, which thus might have served antibody evasion purposes. Indeed, rLCMV/LASΔGlc5 induced a rapid nAb response that clearly exceeded the one to the corresponding WT virus (Fig 2B). In light of the above LCMV-GP data we hypothesized that in the context of Glc5 deficiency, Glc9 might also play a role in delaying and weakening nAb induction to LASV-GP. Indeed, when removing the Glc9 motif in addition to Glc5 on rLCMV/LAS (rLCMV/LASΔGlc5,9) a stepwise increase and acceleration of the nAb response resulted (Fig 2B). In contrast to these clear differences in nAb responses, all rLCMV/LAS variants induced similar NP-specific antibody titers (S2B Fig). Junin vaccine strains lack Glc11, which is present in clinical isolates [39]. Hence we compared nAb induction by rLCMV expressing either the Junin vaccine strain XJ Clone 3 GP (rLCMV/JUN-vacc) or by the analogous virus, in which the consensus motif for Glc11 had been restored (rLCMV/JUN). rLCMV/JUN induced a less potent nAb response than rLCMV/JUN-vacc, again correlating inversely with GP-1 glycan density (Fig 2C).

Importantly, the above results were obtained when assessing serum nAb titers against the very virus used for immunization. Conversely, rLCMVΔGlc9-induced serum antibodies failed to neutralize rLCMV (Fig 2D). Similarly, antibodies elicited by rLCMV/LASΔGlc5,9 neutralized the immunizing virus but failed to detectably neutralize rLCMV/LAS or rLCMV/LASΔGlc5 (Fig 2E). Also rLCMV/JUN-vacc immune sera neutralized preferentially the homologous virus (Fig 2F), analogously to earlier observations in vaccinated monkeys [40]. In line with the clinical efficacy of live-attenuated Junin vaccines [11, 41], rLCMV/JUN-specific neutralizing activity was also detected in rLCMV/JUN-vacc-immune sera but was comparably lower. These findings suggested that partially deglycosylated GP-1 variants elicited an accelerated and more potent nAb response that was, however, largely specific to the glycan-deficient immunogen.

### WT virus-induced antibody responses neutralize preferentially GP-1 variants that lack specific glycans

**Fig 3:**
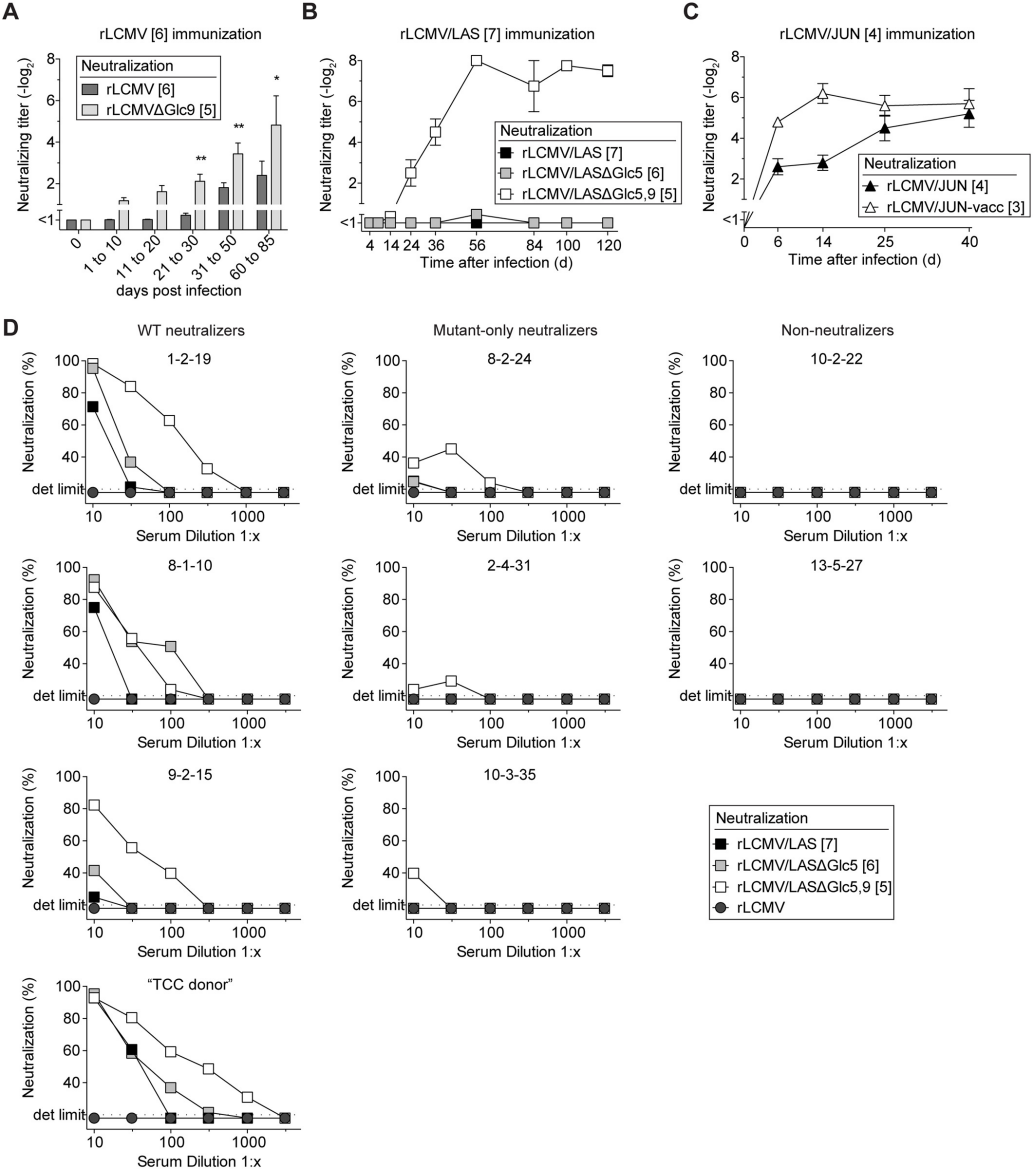
WT virus-induced antibody responses neutralize preferentially GP-1 variants that lack specific glycans. (A-C) We infected C57BL/6 mice i.v. with rLCMV (wt GP, 2x10^5^-4x10^6^ PFU, A), rLCMV/LAS (5x10^5^ PFU, B) or rLCMV/JUN (2x10^5^ PFU, C). Serum samples were collected during the indicated time windows after infection and were tested for their neutralizing capacity against the respective immunizing viruses or their partially deglycosylated variants. (A) Bars represent the mean ± SEM of 23–39 mice per group up to day 50 and of 8 mice per group between days 60–85. A two-way ANOVA followed by Bonferroni’s post-test for multiple comparisons was performed. * *p*<0.05, ** *p*<0.01. Combined data from six independent experiments are shown. (B-C) Symbols represent the mean ± SEM of four to five mice per group. One out of two similar experiments is shown. (A-C) Neutralizing titers were determined in 10-fold (A), 5-fold (B) or 20-fold (C) pre-diluted serum. (D) Convalescent sera of nine individual Lassa patients were tested for neutralizing activity against rLCMV/LAS and its partially deglycosylated variants rLCMV/LASΔGlc5 or rLCMV/LASΔGlc5,9. Neutralization of rLCMV was included as a specificity control. Each graph represents one LASV-convalescent subject. “1-2-19”, “8-2-24”, “10-2-22”, “8-1-10”, “2-4-31”, “13-5-27”, “9-2-15”, “10-3-35” and “TCC donor” represent patient identification codes as previously published [42]. For each virus, the number of N-linked glycosylation motifs in GP-1 is indicated in brackets.

In an inverse approach we assessed whether the antibody response to fully glycosylated wt GPs neutralized the respective partially glycan-deficient variants. Infection with rLCMV induced a late albeit detectable nAb response against itself (Fig 3A). Conversely, rLCMVΔGlc9-neutralizing activity in the same sera was detected earlier and reached higher titers. Even more pronounced, rLCMV/LAS infection elicited a rapid and potent nAb response against the rLCMV/LASΔGlc5,9 variant, but no detectable neutralizing serum activity against rLCMV/LASΔGlc5 or rLCMV/LAS used for infection (Fig 3B). We further corroborated the key contribution of Glc9 in reducing nAb sensitivity of LASV by assessing LASV-GP glycan variants lacking individually either glycosylation motifs 3, 5, 6, 9, 12 or 15 (S3A Fig). Unlike the other mutants tested, rLCMV/LASΔGlc9 was potently neutralized by rLCMV/LAS-immune serum. Finally, the rLCMV/JUN-induced antibody response neutralized rLCMV/JUN-vacc more potently than rLCMV/JUN (Fig 3C). To assess the relevance of these findings for the human immune response to a pathogenic arenavirus, we extended our analysis to LASV-convalescent human sera with known seroreactivity as determined by indirect immunofluorescence [42]. Four out of nine patient sera exhibited detectable neutralizing activity against rLCMV/LAS (Fig 3D). The potency of these “WT neutralizers” sera increased stepwise when tested against the glycan-deficient rLCMV/LASΔGlc5 and rLCMV/LASΔGlc5,9 variants, respectively. In further three out of nine patients (“mutant-only neutralizers”), neutralizing activity was only detectable against rLCMV/LASΔGlc5 and/or rLCMV/LASΔGlc5,9. Two patient sera (“non-neutralizers”) failed to detectably inhibit the infectivity of either virus. These data showed that preferential neutralization of glycan-deficient LASV-GP variants, as observed in rLCMV/LAS-infected mice (Fig 3B), extended to humans infected with wt LASV in the field.

### mAbs neutralize preferentially GP-1 variants that lack specific glycans

**Fig 4:**
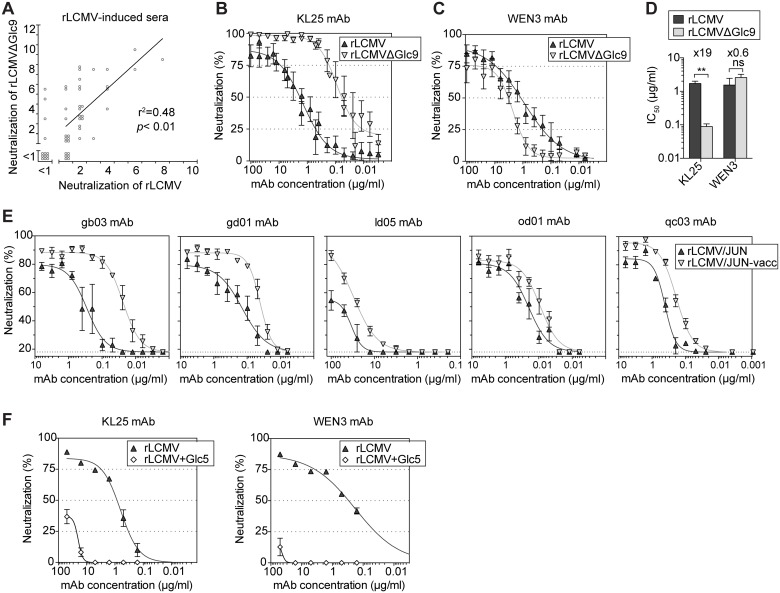
Monoclonal Abs neutralize preferentially GP-1 variants that lack specific glycans. (A) We infected C57BL/6 mice with rLCMV (10^5^−10^6^ PFU) and collected serum after virus clearance (day 40–80). We assessed neutralizing activity against the immunizing rLCMV and against the partially deglycosylated variant rLCMVΔGlc9. Each point represents an individual mouse serum sample. Data from 53 mice in nine independent experiments are summarized. The Pearson’s correlation coefficient and two-tailed *p*-value are indicated. ** *p*<0.01. (B-D) We quantified the neutralization potency of KL25 and WEN3 mAbs against rLCMV (wt GP) and rLCMVΔGlc9, respectively (B-C), and determined half-maximal inhibitory concentrations (IC_50_, D). The mean ±SEM of 7 data points recorded in 5 independent experiments is represented. Unpaired student’s t-tests were used for statistics. ** *p*<0.01. (E) The neutralization potency of 5 mAbs elicited by WT JUNV [45] was individually assessed against rLCMV/JUN (wt GP; four GP-1 glycosylation motifs) or rLCMV/JUN-vacc (three GP-1 glycosylation motifs). Symbols represent the mean ±SEM of 3 replicates per group. One representative out of three experiments is shown. (F) We used KL25 and WEN3 mAbs in neutralization assays against rLCMV (wt GP) and an rLCMV variant in which Glc5 was artificially introduced (rLCMV+Glc5). Symbols represent the mean ±SEM of 3 replicates.

These observations suggested that, both in mice and humans, arenavirus infections elicited serum antibodies that neutralized predominantly glycan-deficient viral variants. In support thereof, a panel of rLCMV-immune mouse sera exhibited a statistically significant correlation between their neutralizing potency against rLCMVΔGlc9 and WT rLCMV (Fig 4A). On average the former activity exceeded the latter one by about four-fold. Analogous observations were made when mice were infected with recombinant LCMV expressing the Armstrong strain GP (rLCMV/ARM) or a variant thereof lacking Glc12 (rLCMV/ARMΔGlc12, corresponding to the formerly described Armstrong 4 isolate [34], S3B Fig). These findings raised the possibility that a proportion of serum antibodies reacted against WT virus and additionally, with higher potency, also neutralized glycan-deficient variant viruses. In support of this hypothesis we found that KL25, a widely used WT LCMV-induced mAb [28], neutralized rLCMVΔGlc9 at roughly 20-fold lower concentration than rLCMV carrying the wt GP (Fig 4B and 4D). Conversely, the IC_50_ of the WEN3 mAb on rLCMV and rLCMVΔGlc9 was not significantly different (Fig 4C and 4D). In concert with this observation, point mutations in KL25 escape variants cluster around Glc9 [43, 44] and don’t affect WEN3 binding or neutralization, suggesting the two mAbs recognize distinct epitopes. An electron microscopic assessment of virion labeling with saturating concentrations of KL25 and WEN3, respectively, indicated that glycoprotein densities on rLCMV and rLCMVΔGlc9 differed by less than 1.5-fold (S4 Fig). This was in line with earlier observations on unimpaired cell surface expression of LCMV-GPΔGlc9 [35] and suggested differential recognition rather than differential availability of the KL25 epitope on rLCMV and rLCMVΔGlc9. Similarly to the behavior of KL25 against LCMV-GP, a panel of WT JUNV-induced mAbs exhibited consistently higher potency against rLCMV/JUN-vacc (3 GP-1 glycans) than against rLCMV/JUN (4 GP-1 glycans; Fig 4E). The relative differences in potency against the two viruses varied, however, between mAbs. Taken together, these observations suggested that neutralizing antibodies, which were induced in response to fully glycosylated wt GPs, exhibited higher potency when specific glycans were removed from the target antigen. In an inverse approach, we introduced Glc5 in LCMV-GP (rLCMV+Glc5) thus mimicking LASV glycosylation. rLCMV+Glc5 was viable [35] but it was ≥100-fold less sensitive to KL25 or WEN3 neutralization than WT virus (Fig 4F), further attesting to the capacity of Glc5 to shield arenaviruses against neutralizing antibodies.

### Facilitated binding of neutralizing mAb to Glc9-deficient LCMV-GP-1

**Fig 5:**
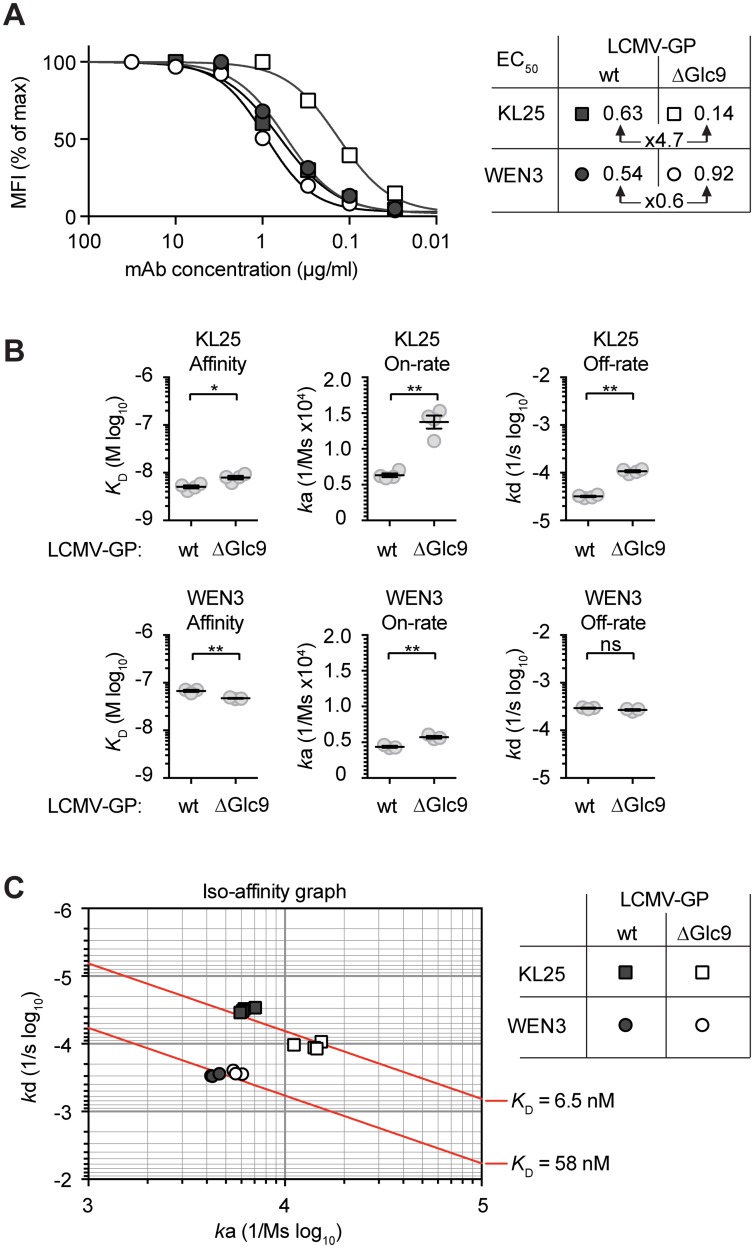
Facilitated binding of neutralizing mAb to Glc9-deficient LCMV-GP-1. (A) We transfected 293T cells with plasmids for expression of either LCMV-GPwt or LCMV-GPΔGlc9. We then incubated these cells with titrated concentrations of KL25 or WEN3 mAbs, and saturation of LCMV-GP binding was characterized by flow cytometry. The mean fluorescence intensity (MFI) is plotted as percentage of maximum staining intensity. Untransfected cells were used for reference. The 50% effective concentrations (EC_50_) are displayed in the chart. One out of two representative experiments is shown. (B-C) Binding kinetics of KL25 and WEN3 Fab fragments to LCMV-GPwt or LCMV-GPΔGlc9 were characterized by surface plasmon resonance (SPR). The association constant (*k*a), dissociation constant (*k*d) and the resulting equilibrium dissociation constant (*K*_D_ = *k*d/*k*a) were determined. Each measurement was done in triplicates for WEN3 and quadruplicates for KL25, respectively. In panel (B) means ± SEM are indicated. * *p*<0.05, ** *p*<0.01 by unpaired student’s *t* tests. (C) *k*a and *k*d values obtained by SPR were plotted on a two-dimensional graph such that identical KD values are located along iso-affinity lines (diagonals).

We hypothesized that facilitated binding was accountable for glycan-dependent differences in neutralization potency of the KL25 mAb. We transfected 293T cells with LCMV-GPwt or LCMV-GPΔGlc9 expression plasmids, respectively, and used flow cytometry to establish saturation curves and resulting EC_50_ values for the Glc9-sensitive KL25 and the Glc9-insensitive WEN3 mAbs. The EC_50_ of KL25 on LCMV-GPΔGlc9 was approximately five-fold lower than on LCMV-GPwt, whereas comparable WEN3 concentrations were required to bind the two LCMV-GP versions (Fig 5A). Thus, higher KL25 occupancy of LCMV-GPΔGlc9 as compared to its wt counterpart contrasted with the indiscriminate behavior of WEN3, matching the neutralization behavior of these mAbs (compare Fig 4B–4D). To further dissect these interactions we performed surface plasmon resonance measurement of KL25 and WEN3 Fab binding to the soluble ectodomains of LCMV-GPΔGlc9 and LCMV-GPwt. Counter to expectations based on neutralization sensitivity (Fig 4B), the overall affinity of KL25 Fab binding to LCMV-GPΔGlc9 was modestly lower than its binding to LCMV-GPwt (i.e. higher *K*_D_ = *k*d/*k*a; Figs 5B and S5). This was due to a higher off-rate (*k*d) that partially counterbalanced an elevated on-rate (*k*a). Thus, like for mAbs against members from other viral families [46, 47], the higher on-rate of the KL25 Fab on LCMV-GPΔGlc9 than on LCMV-GPwt represented the best correlate of increased neutralizing potency of the dimeric full length mAb. Conversely, WEN3 affinity was slightly lower on LCMV-GPΔGlc9 than on LCMV-GPwt, and on-rate differed only modestly, which was in agreement with virtually identical neutralizing potency and binding in flow cytometry. Iso-affinity plots illustrated the non-discriminative binding behavior of WEN3, which contrasted with the differential on-rate but comparable overall affinity of KL25 for the wt and Glc9-deficient GP variants, respectively (Fig 5C). Taken together, the reduction in antibody on-rate offered a mechanistic explanation how Glc9 shielded LCMV-GP against antibody neutralization.

### Structural model of LASV GP-1 glycosylation

**Fig 6:**
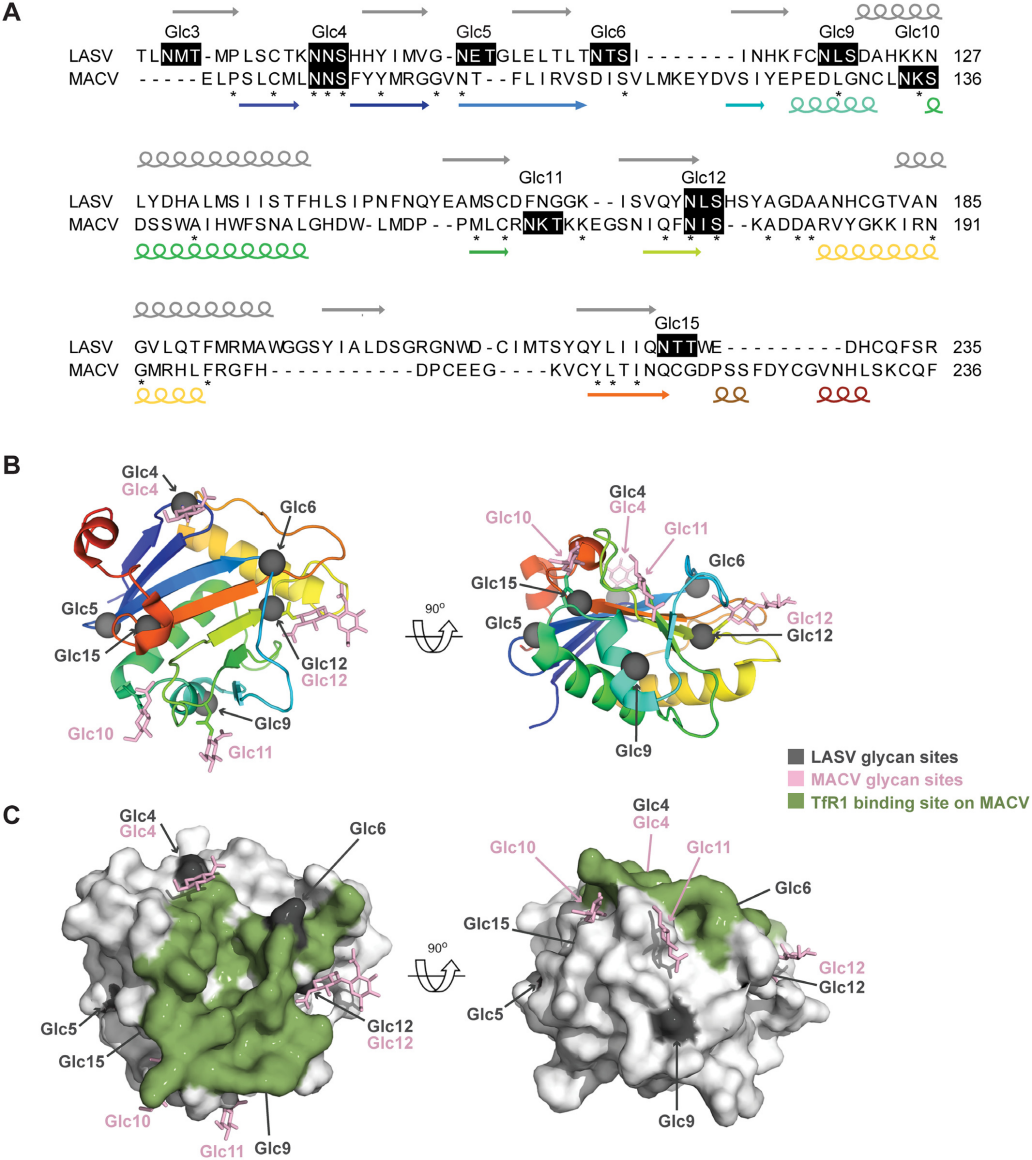
Mapping LASV GP-1 N-linked glycan sequons onto the structure of MACV GP-1. (A) The predicted secondary structure composition of LASV GP-1 (above the sequence) was compared to the secondary structure observed in the crystal structure of MACV GP-1 (below the sequence, [48]). Arrows represent beta-sheets and spirals represent helices, the rainbow code corresponds to panel B. Stars indicate identical residues. The number and position of predicted N-linked glycans are indicated above black shaded sequons. The alignment and annotations were extracted from the arenavirus GP-1 sequences alignment presented in S1 Fig. (B) Cartoon diagram of MACV GP-1 colored as a rainbow ramped from blue (N-terminus) to red (C-terminus). Sites of N-linked glycosylation observed in the MACV GP-1 crystal structure are annotated and shown as pink sticks. The locations of putative N-linked glycosylation sequons from LASV GP-1 were mapped by sequence alignment (S1 Fig) and are shown as grey spheres. (C) Surface of MACV GP-1 shown in van der Waals surface representation. Residues on MACV GP-1 which form contacts with human TfR1 are colored green. N-linked glycan sites and sequons are colored as in panel B.

The only arenavirus pre-fusion GP-1 structure that has been resolved is that from Machupo virus [48, 49]. Despite the low sequence homology of MACV GP-1 with LASV GP-1, secondary structure predictions (Fig 6A) indicated that their core folds were conserved, and a web-based algorithm [50] calculated 100% confidence for structural homology. This prompted us to map the location of LASV GP-1 N-linked glycans onto the MACV GP-1 surface (Fig 6B). Glc5, Glc9 and Glc12, which apparently reduce neutralization sensitivity of Old World arenaviruses, all projected onto solvent-exposed loops outside the receptor-binding footprint on MACV (Fig 6C). A limitation of this model consists in the fact that clade B viruses, such as MACV, utilize transferrin receptor 1 for entry [51] whereas alpha-dystroglycan serves as receptor for the Old World arenaviruses LASV and LCMV [52]. Receptor binding sites for the latter two viruses have not yet been mapped. Nevertheless, the model supports the mechanistic postulate that Glc5, Glc9 and Glc12 serve to shield arenaviruses against antibodies by reducing their access to highly immunogenic protein loops on the GP-1 surface. The clustering of KL25 mAb escape mutations around Glc9 [43, 44] is also in line with this concept but additional structural information on arenavirus envelope GPs will be required to formally test these assumptions.

### Glc9-mediated nAb evasion promotes protracted LCMV infection in mice

**Fig 7:**
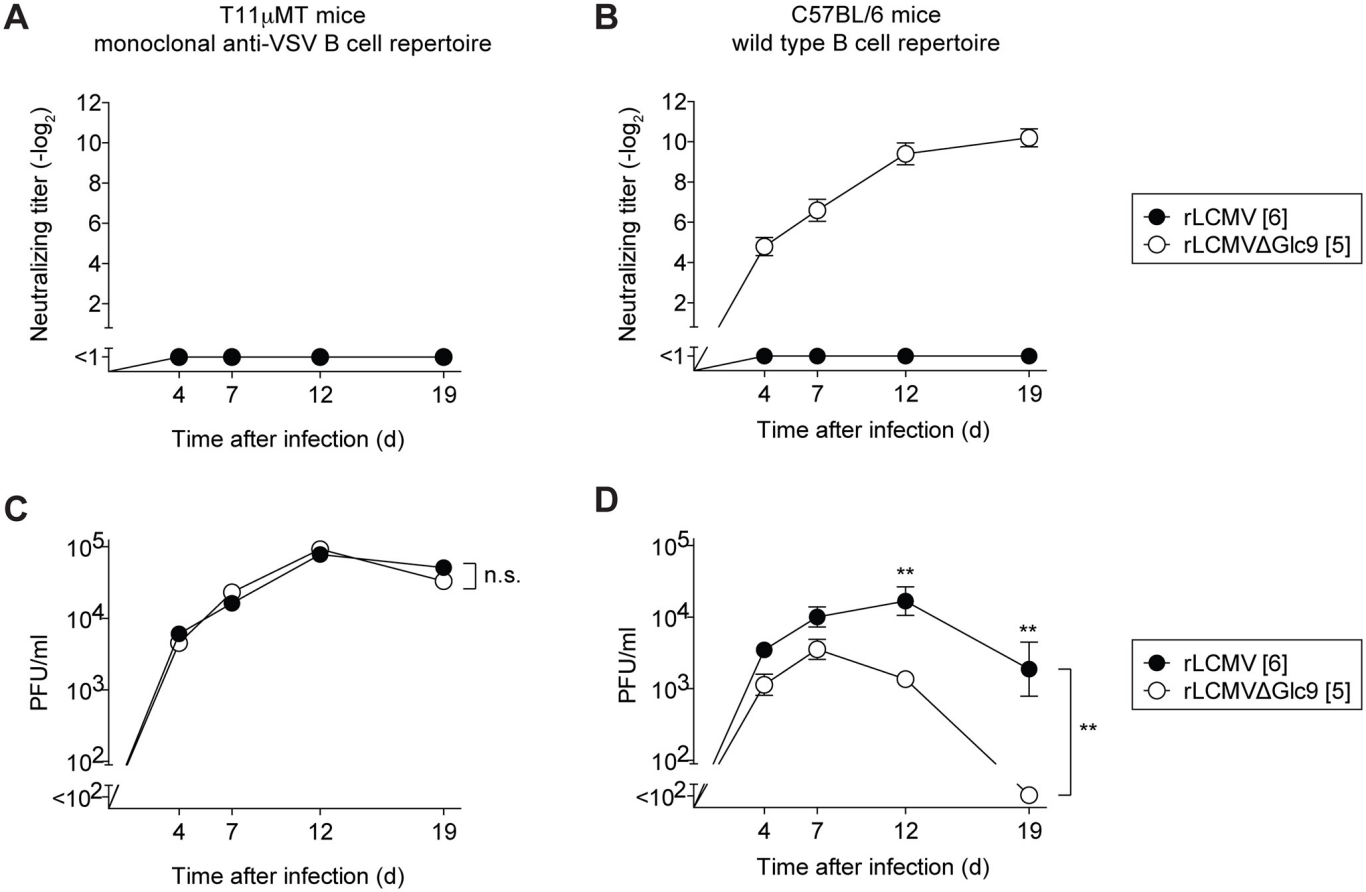
Glc9-mediated nAb evasion promotes protracted LCMV infection in mice. (A-D) We infected T11μMT and C57BL/6 mice i.v. with 4x10^6^ PFU of rLCMV or rLCMVΔGlc9. Blood and serum samples were collected over time. (A-B) Serum samples were tested for their neutralizing capacity against the respective immunizing viruses. Symbols represent the mean ± SEM of four to five mice per group. Neutralizing titers were determined in 10-fold pre-diluted serum. (C-D) Viremia was measured in whole blood samples. Symbols represent the mean ± SEM of four to five mice per group. Differences between rLCMV and rLCMVΔGlc9 viral loads were assessed by two-way ANOVA, followed by a Bonferroni post-test for individual time points if the F-test of ANOVA indicated statistically significant differences. ** *p*<0.01.

nAb responses not only protect against viral reinfection but can also help resolving primary infection [26, 53]. Hence we tested the possibility that the arenavirus glycan shield impeded efficient virus control by promoting nAb evasion. For this we exploited T11μMT mice, which mount normal CD4^+^ and CD8^+^ T cell responses to LCMV [26], but have a quasi-monoclonal B cell repertoire recognizing virtually exclusively the LCMV-unrelated glycoprotein of vesicular stomatitis virus. Accordingly, T11μMT mice failed to mount nAb responses when infected with either rLCMVΔGlc9 or WT rLCMV (Fig 7A). In contrast, wild type mice mounted a rapid and potent nAb response against rLCMVΔGlc9 but not against fully glycosylated rLCMV, as expected (Fig 7B). Therefore the comparison of viral loads in these two congenic strains of mice allowed us to directly assess the impact of the rapid nAb response on rLCMVΔGlc9 control. In concert with identical growth of rLCMVΔGlc9 and rLCMV in cell culture (S6 Fig), the two viruses persisted at indistinguishable levels in the blood of T11μMT mice throughout the observation period of 19 days (Fig 7C). In contrast, rLCMVΔGlc9 was cleared from the blood of C57BL/6 wt mice by day 19, whereas the glycan-shielded rLCMV virus persisted (Fig 7D). This protracted course of infection was expected for the LCMV strain Clone 13-based viruses used in our experiments [54, 55]. Viral loads in blood of rLCMVΔGlc9- and rLCMV-infected C57BL/6 mice were significantly different from day 12 onwards, which was in line with the early onset of the nAb response. Altogether, this demonstrated that glycan-mediated nAb evasion promotes protracted LCMV infection.

## Discussion

Glycan shielding of arenavirus GPs provides an explanation for the consistent failure to induce potent LASV-specific antibody immunity by either vaccination or natural infection [14, 15, 17, 18, 56–59]. In both circumstances, specific ELISA titers were high while neutralizing activity remained modest at best [56–58]. In line with these observations, our data suggest that glycosylation does not primarily prevent GP-1-specific antibody induction, but it impairs the capacity of these antibodies to neutralize. This shielding mechanism we propose differs from previous concepts such as the supposed “hole” in the arenavirus GP-1-specific B cell repertoire [25]. Neither would the arenavirus GP-1 represent the equivalent of an “immunologically silent face” in HIV-1 [60], i.e. GP-1 does not seem to lack immunogenicity owing to glycan resemblance to “self”. Our observations are more reminiscent of the “glycan shield” concept for HIV-1 [3], proposing that glycans impair antibody access to neutralizing epitopes on gp120. In stark contrast to HIV-1, however, the available sequence data suggest that arenavirus GP-1 glycans are invariable between isolates. Conversely, the difference in glycan density between the GP-1 of JUNV and LASV represents a likely reason why the excellent therapeutic success of convalescent serum in Argentine hemorrhagic fever does not find a parallel in Lassa fever [17, 21]. The presence of comparably fewer glycans in JUNV-GP-1 also is likely to facilitate the induction of antibody-mediated protection by the live-attenuated JUNV vaccine Candid#1 [11, 41]. In contrast, our data indicate that glycan-deficient LASV-GPs as immunogens will not overcome these structural hurdles.

Removal of Glc9 from LCMV-GP (LCMV-GPΔGlc9) increased the association rate of the neutralizing mAb KL25, thus augmenting the antibody’s ability to neutralize in spite of its slightly lower affinity for this deglycosylated target antigen. This interpretation is in line with the result from large-scale mutagenesis studies on the therapeutic RSV antibody Palivizumab, demonstrating that a fairly modest increase in association rate can translate into a considerably heightened neutralizing potency. Conversely, dissociation rates were found of comparably minor impact owing to the dimeric nature of IgG binding [46]. Analogously mathematical models predict Ab on-rate as a major determinant of HIV-neutralizing Ab potency [47]. We hypothesize therefore that key glycans such as Glc9 and Glc5 in LASV-GP-1 shield the virus against nAbs by reducing their access to neutralizing epitopes. Glycan-mediated stabilization of a distinct poorly accessible pre-fusion conformation represents an alternative and not mutually exclusive mechanism [61].

Effective prevention of Lassa fever remains a priority in West Africa where LASV is endemic. Additionally, a vaccine would allow for the timely containment of potential future outbreaks and for the protection of healthcare workers. As illustrated by the recent Ebola epidemic, which has ravaged the same geographic area [62], viral hemorrhagic fevers can rapidly emerge to a global health concern. Hence, international efforts at developing a LASV vaccine should be intensified up to the level of human clinical trials [12]. Amongst a larger number of candidates (reviewed in [12, 63]), the LASV-related apathogenic arenavirus Mopeia (MOPV), a chimeric LASV/MOPV reassortant virus (ML29), recombinant vaccinia viruses expressing LASV structural proteins and a replicating vesicular stomatitis virus expressing LAS-GP have shown safety and efficacy in non-human primate models [56–59]. Intriguingly, the protective efficacy of all of these vaccines has been accredited to cell-mediated immunity. Also for MOPV, a high level of sequence similarity to LASV (76%, 74%, 57% and 58% for GP, NP, L and Z, respectively), and the induction of LASV-specific T cell responses in MOPV-infected mice [64] support this interpretation. As a second example of heterologous arenavirus immunity, the attenuated Junin vaccine strain XJ clone 3 induced negligible MACV-specific nAb titers but protected against disease upon MACV challenge [65]. High sequence similarity (69%, 88%, 73% and 76% for GP, NP, L and Z, respectively) as a basis for cross-protective T cell immunity seems a likely mechanism, but an accelerated nAb response upon challenge [65] could also have contributed to MACV control. In light of the present findings, modest glycan density on MACV-GP-1 as compared to LASV-GP-1 may have facilitated this response (compare Fig 1B).

The use of life-attenuated or replicating vectored vaccines can be associated with significant reactogenicity as well as with anticipated (infancy, pregnancy) or unexpected safety issues [66, 67]. These constraints are of lesser concern in an outbreak control setting, and may also be acceptable if protection requires potent T cell responses, notably of the CD8^+^ subset. Conversely, if nAbs could be exploited as effectors of protection, inactivated or subunit vaccines might be preferable for population-wide vaccination campaigns as will be needed to control endemic Lassa fever in West Africa. In order for a LASV vaccine to reproduce the success of the numerous antibody-based vaccines in clinical use today [1, 2], a profound understanding of the hurdles on this path is of paramount importance. As recently exemplified for respiratory syncytial virus (RSV), epitope-focused scaffold-based vaccine design can generate artificial vaccine antigens for challenging antibody targets [68]. A detailed mechanistic understanding of LASV nAb evasion will represent an essential basis to generate analogous scaffold-based approaches for this virus. Additional structural information on arenavirus GPs [48, 49] including analyses of their interactions with nAbs will also be required. Still, it remains uncertain whether scaffold-embedded epitopes as immunogens can induce potent nAb responses against glycan-shielded viral epitopes [69]. Hence, our results can also be taken as a rationale to argue that for densely glycosylated arenaviruses such as LASV, nAb-based vaccination may not be feasible. By providing this mechanistic explanation, our data will help justifying the clinical use of more reactogenic vaccine delivery strategies such as life-attenuated and replicating vectored vaccines, which can induce potent T cell-based protection [56–59].

For Lassa fever, high viral loads are a predictor of lethal outcome [13]. Augmented and prolonged viremia due to glycan-mediated nAb evasion (Fig 7) may thus suggest that the envelope glycan shield represents an arenavirus virulence factor. For Junin virus, reverse genetic mapping studies have been conducted both in suckling mice and guinea pigs, and have unanimously identified an attenuating mutation in the GP-2 transmembrane domain of the Candid #1 vaccine strain, which reduces virion infectivity [39, 70]. Unlike in suckling mice, however, the guinea pig model has provided evidence for at least one additional attenuating mutation in GP, with impact on viral dissemination and disease [70]. It seems tempting to speculate that Glc11 deficiency of Candid#1, which we show can facilitate antibody neutralization, may have contributed to the vaccine’s attenuated phenotype in guinea pigs. The failure to detect a putative Glc11 effect in suckling mice [39] could have been due to these young animals’ immunological immaturity, which entails reduced antibody responsiveness [71]. In addition, the more rapid disease course in mice (~10 days) as compared to guinea pigs (~18 days) may have outpaced nAb effects [53, 70, 72]. Alternatively, cell-mediated immunity may control primary Candid#1 infection largely independently of nAb responses [53].

In summary, our study shows that specific GP-1 glycans shield the arenavirus envelope against efficient antibody neutralization, thus limiting the protective capacity of humoral immune defense and promoting protracted infection. This lends a novel perspective on these viruses’ immune evasion strategies and provides strategic guidance for LASV vaccine development.

## Materials and Methods

### Mice

C57BL/6 mice were bred at the Institute for Laboratory Animal Sciences of the University of Zurich or were purchased from Charles River Laboratories. Animal experiments were performed at the Universities of Zurich, Geneva and Basel. Experimental groups were sex and age-matched.

### Human sera

Anonymised human sera were obtained from a serum bank at the University of Marburg, Germany. They originate from a highly LASV-endemic area of Guinea and were identified as seropositive for LASV by immunofluorescence. They correspond to the previously characterized sera from 1999 [42].

### Recombinant viruses and glycosylation mutants

LCMV clone 13 expressing either the LCMV WE strain glycoprotein (referred to as rLCMV WT herein), heterologous arenavirus GPs or glycosylation variants thereof were generated from cDNA by reverse genetic techniques [73]. J. C. de la Torre generously provided a cDNA of the Lassa virus strain Josiah GP. Reverse transcribed virion RNAs of Machupo and Guanarito virus were generously provided by R. Charrel. The GPs of the Junin vaccine strain XJ clone 3, of Amapari and Tamiami virus were RT-PCR cloned from virion RNA. The viruses were kindly provided by R. Zinkernagel. To substitute the GP ORF in the LCMV S segment cDNA for heterologous GP cDNAs, a PCR cloning strategy was utilized as previously outlined in detail [74]. N-linked glycosylation sites were deleted by either a two-way PCR or a circular PCR strategy, mutating the N-X-S/T motif to Q-X-S/T based on a double-nucleotide change in the respective codon. Additional glycosylation sites were introduced by analogous procedures. The sequences of all cDNAs used for virus rescue have been validated by DNA sequencing. Oligonucleotide primer sequences are available from the authors upon request.

### Virus titration, neutralization assays and monoclonal antibodies

Viruses to be used for neutralization assays were generally grown on BHK-21. Viruses for immunization of mice were grown on BHK-21 or 293T-GP cells [75]. Vero cells were used for work with rLCMV/TAC. BHK-21, 293T and Vero cells were obtained from the American Type Culture Collection (ATCC). All viruses were titrated as previously described [75, 76]. The neutralizing capacity of mAbs and immune serum was tested in immunofocus reduction assays [27, 76]. Sera were typically pre-diluted 1/8 or 1/10, followed by serial two-fold dilution steps, and were tested against a constant amount of virus. Neutralizing titers of mouse serum are expressed as the serum dilution yielding 50% immunofocus reduction. To reflect this assay setup in the figures, neutralizing titers are reported as negative log_2_ values, which must be multiplied by the pre-dilution factor given in each figure legend. For example, a neutralizing titer of 3 determined in 8-fold pre-diluted serum indicates a 50% neutralizing titer at a serum dilution of 1:32. For a more precise assessment of the potency of human LASV-convalescent sera and mAbs, the number of foci at any given serum dilution or antibody concentration was expressed in percent of the average number of foci obtained in the absence of serum or mAb. To obtain a precise IC_50_ value for mAbs, the latter type of measurement was automated for high throughput using an immunospot reader (Cellular Technology Ltd.), and neutralization curves were drawn using Graphpad Prism software. The LCMV monoclonal antibodies have been described [27, 28]. JUNV-specific antibodies [45] were generously provided by the Biodefense and Emerging Infections Research Resources Repository (BEI Resources; catalog numbers: GB03 (NR-2564), GD01 (NR-2565), LD05 (NR-2569), OD01 (NR-2567), QC03 (NR-2566)). Contributors to the BEI catalog were NIH Biodefense and Emerging Infections Research Resources Repository and BEI Resources.

### LCMV NP-specific ELISA

To measure LCMV-specific IgG titers, 96-well plates were coated with 100 μl of recombinant, bacterially expressed LCMV-NP at 3 μg/ml in sodium carbonate buffer (pH 9.6). Plates were blocked for 2h with 5% milk in PBS-Tween 0.05% (PBS-T-milk). In a parallel 96-well plate serum samples were prediluted 1:100 in PBS-T-milk, and threefold dilution series were performed. 100 μl of the diluted serum samples was then transferred to the NP-coated plate for 1h. Finally, the plates were incubated for 1h with HRP-coupled goat anti-mouse IgG Ab (Jackson 115-035-062) diluted 1:1’000 in PBS-T-milk. HRP was detected by addition of ABTS color reaction. All steps were carried out at room temperature. Plates were washed three times with PBS-T between each step. NP-specific IgG titers were defined as the log_3_ dilution resulting in an optical density at 450 nm that was twofold above background.

### Flow cytometric assessment of antibody binding

To assess KL25 and WEN3 mAb binding to the native membrane-bound form of LCMV-GP, 293T cells were transfected with LCMV-GPwt or ΔGlc9 using saturating plasmid amounts. 48 hours later, we harvested the cells and stained them with titrated concentrations of KL25 or WEN-3 mAb for 5 min at RT followed by detection with PE-conjugated goat anti-mouse IgG. The fluorescence signal was measured on an LSR Fortessa flow cytometer (BD) and was analyzed using FlowJo software.

### Surface Plasmon Resonance (SPR) on recombinant soluble LCMV-GP

For use in SPR assays, the ectodomain of the LCMV-GPwt (WE strain, aa 1–430) and the respective Glc9 mutant version were C-terminally fused to streptag II (SA-WSHPQFEK(GGGS)2GGSAWSHPQFEK; Twin-Strep-tag, IBA GmbH, Germany) and were expressed in transiently transfected 293T cells. The protein was purified for SPR assays using Strep-tactin purification columns according to the manufacturer’s instructions (IBA GmbH, Germany). KL25 and WEN3 Fabs were obtained by recombinant expression and enzymatic cleavage, respectively. Affinity and kinetics of Fab binding were determined on a Biacore 2000 (GE Healthcare, Uppsala, Sweden). A CM5 sensor chip (GE Healthcare, Uppsala, Sweden) was covalently coupled with the StrepMAB-Immo antibody (IBA BioTAGnology, St. Louis, MO) by amine coupling. The surface was activated for 7 min at 10 μL/min with a mix 1:1 containing EDC and NHS solutions to final concentrations of 200 and 100 nM respectively. The StrepMAB-Immo was diluted in 10 mM acetate buffer at pH 5.0 and injected at 10 μL/min for 7 min. Unused activated chip surface was blocked by injecting 1 M ethanolamine for 7 min. This process resulted in the immobilization of StrepMAB-Immo antibody at densities ranging from 5000 to 10000 RU. Then, soluble LCMV-GP (wt or Glc9 mutant) was injected for 5 min at a concentration of 50 μg/mL and a flow rate of 5 μL/min, leading to capture levels between 800 and 1500 RU. Kinetics were performed at 25°C, in HBS EP buffer (GE Healthcare, Uppsala, Sweden), at a flow rate of 30 μL/min. KL25 and WEN3 Fabs were injected for 5 min in duplicate and randomly at five and six decreasing concentrations, starting from 500nM and 1000nM, respectively. The dissociation phase was monitored for 30 min. Regeneration was assessed using a 10 mM glycine pH 1.5 solution injected for 3 min. Curves were fitted according to the 1:1 Langmuir binding model and using the BIAevaluation 4.1.1 software (GE Healthcare, Uppsala, Sweden). A double referencing was applied for analysis to subtract buffer signal drift on coated surface and unspecific background signal on a reference channel. All experiments were performed in triplicates.

### Electron microscopy

BHK-21 cells were infected with either rLCMV or rLCMVΔGlc9 at MOI 0.1. 40 h later, the cells were fixed for 60 min at RT in 0.1 M phosphate buffer (pH 7.4) containing 2% paraformaldehyde and 0.02% glutaraldehyde. After washing, the cells were scraped off the culture dishes, embedded in 12% gelatin, infused with 2.3 M sucrose, frozen in liquid nitrogen, and sectioned with a EMFCS ultracryomicrotome (Leica). Ultrathin sections were immunostained for 15 h with either KL25 or WEN-3 mAb at a saturating concentration of 100 μg/ml, followed by a 20 min RT incubation with Protein A-coated 10 nm gold particles [77]. Cryosections were screened and photographed using a CM10 electron microscope (Philips, Eindhoven, The Netherlands). For the evaluation of GP labeling density on virions, 160–180 cells were photographed at 21,000-fold magnification and the number of gold particles per virion was manually counted.

### Sequence alignments and structural model of LASV N-linked glycans in GP-1

Amino acid sequence alignments and automatic N-X-S/T motif searches were performed using the Jalview software [78]. The secondary structure of LASV GP-1 was predicted with NPS@ [79]. Fold prediction was performed using the Phyre2 fold prediction tool [50]. The MACV GP-1 structure was previously published (PDB accession number 2WFO [48]) and residues forming contacts with human TfR1 were determined with the PISA EBI server [80] using PDB accession number 3KAS [49]. Protein sequence similarities of LASV/MOPV and JUNV/MACV described in the discussion section were calculated online with BLASTp, using comparison of two protein sequences [81].

### Statistics

For statistical analysis, the GraphPad Prism software (version 5.04, GraphPad Software, San Diego, California) was used throughout. Titers values were log-converted to obtain a near-normal distribution. To assess significant differences between single measurements of 2 groups we used two-tailed Student’s *t* tests. Differences between multiple measurements of 2 or more groups were assessed by two-way ANOVA followed by multiple t tests with Bonferroni adjustment for multiple comparisons if the F test of ANOVA indicated statistically significant differences. To analyze correlations, linear regression was performed and the Pearson’s correlation coefficient as well as a two-tailed *p*-value were calculated. *P*-values <0.05 were considered statistically significant (*), *p*<0.01 was considered highly significant (**) and *p*>0.05 was considered not statistically significant (ns).

### Ethics statement

Animal experiments were approved by the Cantonal Veterinary Office of the Canton of Zurich (permission 176/2005), the Direction Générale de la Santé (permissions 1005/3312/2 and 1005/3312/2-R) of the Canton of Geneva, and the Cantonal Veterinary Office of the Canton of Basel (permission 24257/2666), respectively. All animal experiments were performed in accordance with the Swiss law for animal protection. The measurements of LASV-nAbs in anonymised human sera were performed with ethical approval by the Ethik-Kommission des Kantons Zürich (KEK, Ref. Nr.: StV 49–2006).

### Accession numbers

The Genbank accession numbers for genes and proteins mentioned in this study are shown below in parentheses. LUJV (FJ952384), DANV (EU136038), LCMV-WE (AJ297484), LCMV-ARM (AY847350), LASV (J04324), MOBV (AY342390), MOPV (AY772170), MORV (EU914103), IPPYV (DQ328877), FLEV (AF512831), ALLV (AY012687), PARV (AF485261), PIRV (AF277659), PICV (K02734), OLVV (U34248), LATV (AF485259), JUNV-vacc (HQ126699), JUNV (AY358023), TCRV (KP159416), MACV (AY619643), AMAV (AF512834), GTOV (AF485258), CPXV (AF512832), SABV (U41071), CHP (EU260463), BCNV (AY924391), CATV (DQ865244), NAAV (EU123329), SKTV (EU123328), TAMV (AF512828), WWAV (AF228063). The arenaviruses’ full names corresponding to the above acronyms can be found in Table 1.

## Supporting Information

S1 Fig Alignment of various arenavirus GP-1 sequences. A sequence alignment was performed using Jalview [78]. All predicted N-glycosylation sites (NX[S/T] motifs) are highlighted (black shaded) and the corresponding glycans (Glc) are numbered from 1 to 15. Blue shaded amino acids denote a high degree of conservation amongst many arenaviruses. The five C-terminal amino acids correspond to the SKI-1/S1P core recognition motif between GP-1 and GP-2. OW: Old World arenaviruses, NW: New World arenaviruses. For abbreviations of viruses and Genbank accession numbers, see Table 1.
(TIF)

S2 Fig NP-specific antibody responses to rLCMV/GP variant viruses. (A, B) Mice were infected i.v. with 4x10^6^ PFU (A) or 5x10^5^ PFU (B) of the indicated rLCMV/GP variants. LCMV-NP specific IgG titers were determined in 100-fold pre-diluted serum on d25 (A) or d42 (B) after infection. Individual data points and the mean ±SEM of four to five mice per group are shown.
(TIF)

S3 Fig Key role of LASV Glc9 in preventing antibody neutralization and direct correlation between neutralizing potency of serum antibodies against rLCMV/ARM and its glycan-deficient variant rLCMV/ARMΔGlc12. (A) Mice were primed and boosted i.v. with 10^4^ PFU of rLCMV/LAS (wt GP) on day 0 and 59. Serum was collected on day 105 and tested in neutralization assays against rLCMV/LAS-GP variants lacking either the glycosylation motifs 3, 5, 6, 9, 12 or 15. Neutralizing titers were determined in 8-fold pre-diluted serum. Of note, we failed to recover rLCMV/LAS-GPΔGlc9, which matches analogous observations with LCMV-GP mutant viruses [35], thus corroborating a supposed structural key role of the highly conserved Glc4 in arenavirus GPs (compare Table 1). (B) We infected C57BL/6 mice with 4x10^6^ PFU of rLCMV/ARM i.v. and collected serum samples in the time window between day 60 to 67. We assessed their neutralizing activity against the immunizing rLCMV/ARM and its partially deglycosylated variant rLCMV/ARMΔGlc12, respectively. The number of N-linked glycosylation motifs in GP-1 of each variant is indicated in brackets. Each data point represents a serum sample from an individual mouse. Combined data from 29 mice in five different experiments are shown, demonstrating a positive correlation between rLCMV/ARM- and rLCMV/ARMΔGlc12-neutralizing activity. The Pearson’s correlation coefficient and two-tailed *p*-value are indicated. ** *p*<0.01.
(TIF)

S4 Fig Comparable incorporation density of LCMV-GPwt and LCMV-GPΔGlc9 in virions. We infected BHK-21 cells with either rLCMV WT or rLCMVΔGlc9 for 48 hours and determined GP incorporation density by pre-embedding electron microscopy on budding virions. Bound KL25 or WEN3 mAbs were detected using a gold-coupled secondary antibody. (A) Representative electron micrographs showing budding virions with immunogold-labeled GP. Scale bar: 100 nm. (B) For both, KL25 and WEN3, the numbers of gold particles per virion were counted. Bars represent the mean +SEM of the following numbers of virions assessed in each staining combination. KL25 on rLCMV WT n = 204, KL25 on rLCMVΔGlc9 n = 129, WEN3 on rLCMV WT n = 88, WEN3 on rLCMVΔGlc9 n = 71.
(TIF)

S5 Fig Increased association rate of KL25 Fab binding to Glc9-deficient LCMV-GP-1. Binding kinetics of KL25 and WEN3 Fabs on LCMV-GPwt and ΔGlc9 as determined by surface plasmon resonance (SPR). The binding curves (colored lines) were globally fitted to a 1:1 Langmuir binding model (black line). Fabs were used at titrated concentrations as indicated on the graphs. For each condition, one representative binding curve out of three to four replicate measurements is shown. The corresponding binding constants are plotted in Fig 5.
(TIF)

S6 Fig Identical growth of rLCMVΔGlc9 and rLCMV in cell culture. BHK-21 cells (5x10^5^ per M6 well) were infected with the indicated viruses at a multiplicity of infection of 0.01 and infectious virus in the supernatant was measured at the indicated time points. Symbols indicate the mean ±SD of three tissue culture wells (error bars project into the symbols).
(TIF)
