# Supplementary figures and images for: Arenavirus Glycan Shield Promotes Neutralizing Antibody Evasion and Protracted Infection
Source: PLoS Pathog. 2015 Nov 20;11(11):e1005276. doi: 10.1371/journal.ppat.1005276 (PMC4654586; doi:10.1371/journal.ppat.1005276)

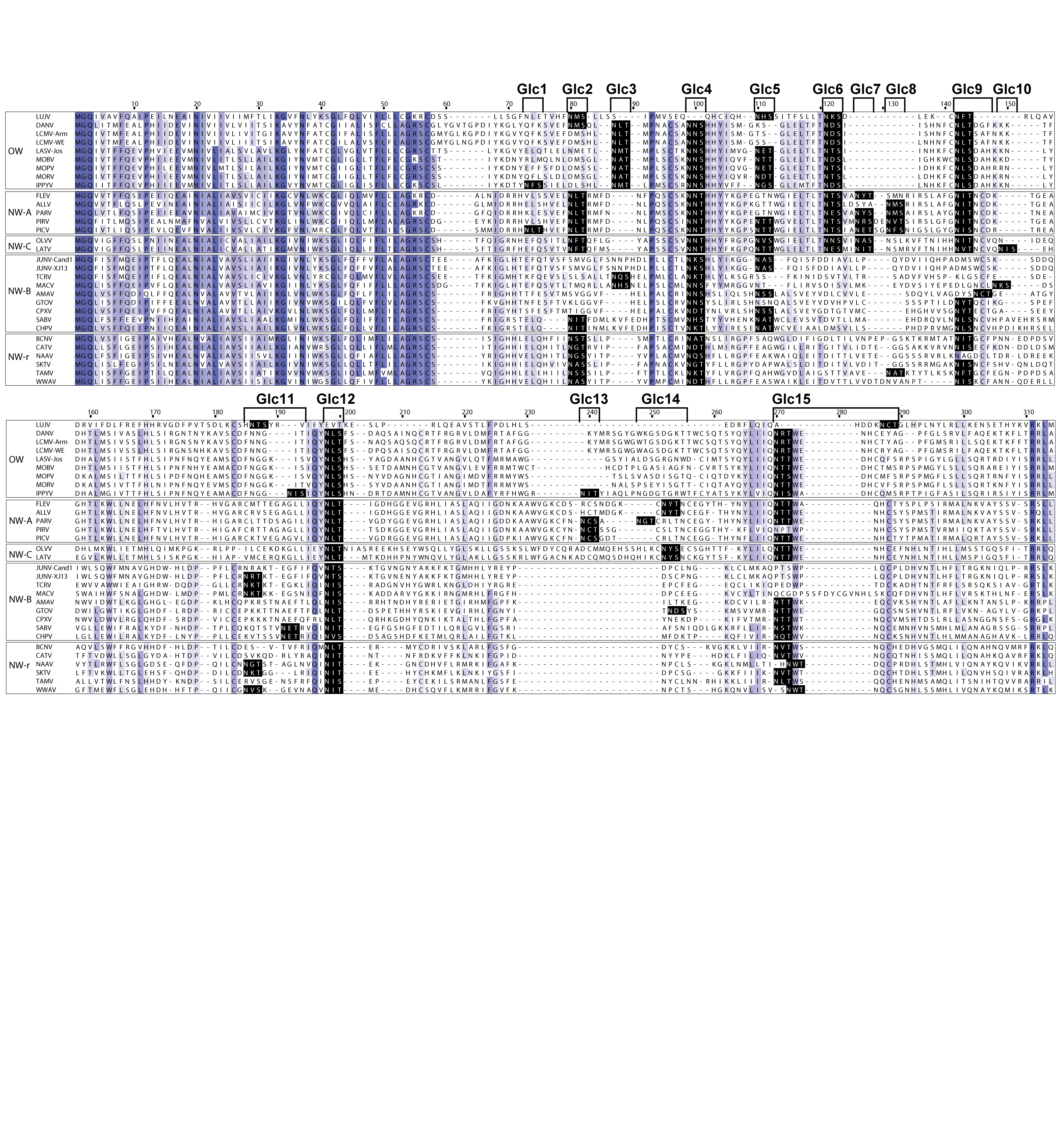

Supplement: S1 Fig — A sequence alignment was performed using Jalview [78]. All predicted N-glycosylation sites (NX[S/T] motifs) are highlighted (black shaded) and the corresponding glycans (Glc) are numbered from 1 to 15. Blue shaded amino acids denote a high degree of conservation amongst many arenaviruses. The five C-terminal amino acids correspond to the SKI-1/S1P core recognition motif between GP-1 and GP-2. OW: Old World arenaviruses, NW: New World arenaviruses. For abbreviations of viruses and Genbank accession numbers, see Table 1. (TIF) [file ppat.1005276.s001.tif]

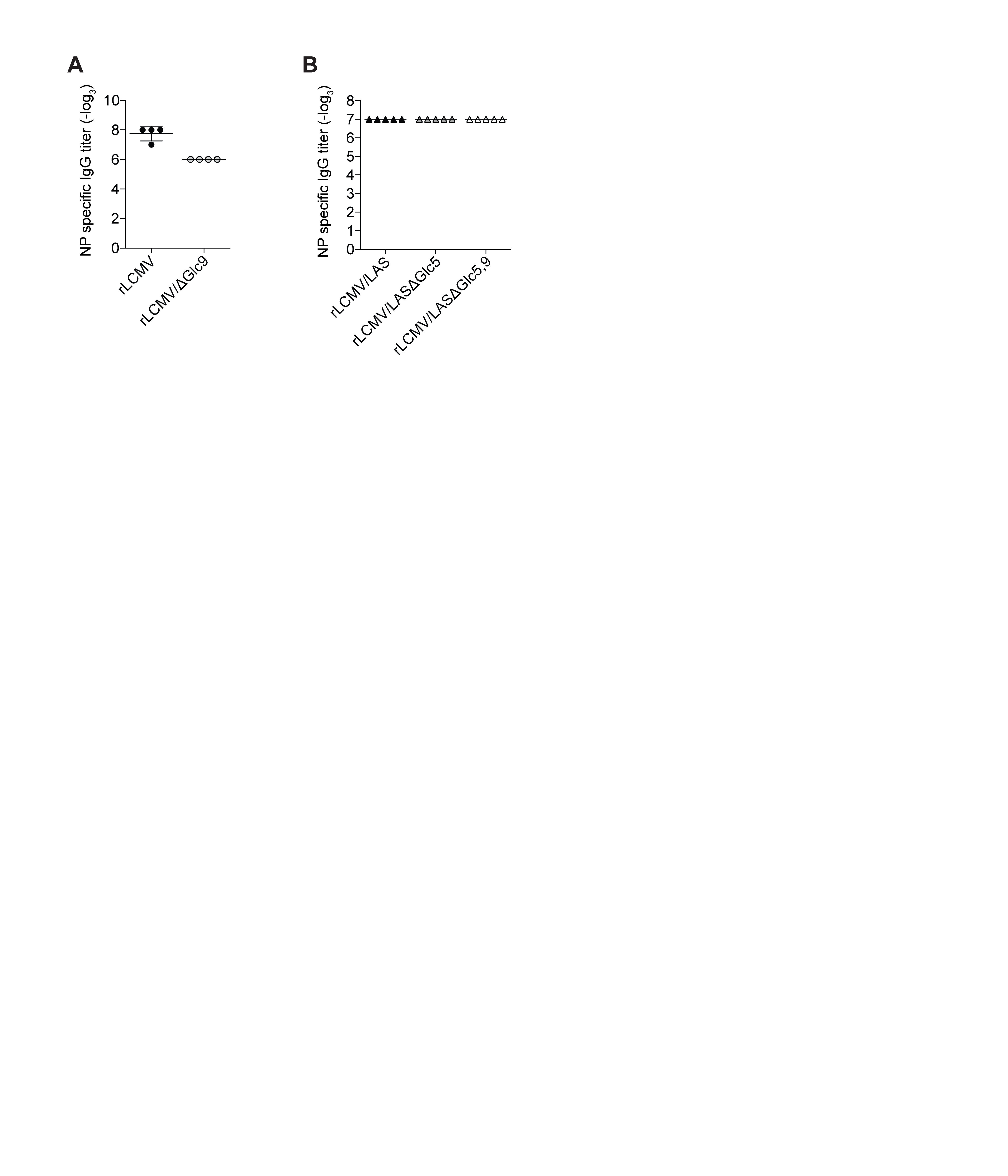

Supplement: S2 Fig — (A, B) Mice were infected i.v. with 4x10^6^ PFU (A) or 5x10^5^ PFU (B) of the indicated rLCMV/GP variants. LCMV-NP specific IgG titers were determined in 100-fold pre-diluted serum on d25 (A) or d42 (B) after infection. Individual data points and the mean ±SEM of four to five mice per group are shown. (TIF) [file ppat.1005276.s002.tif]

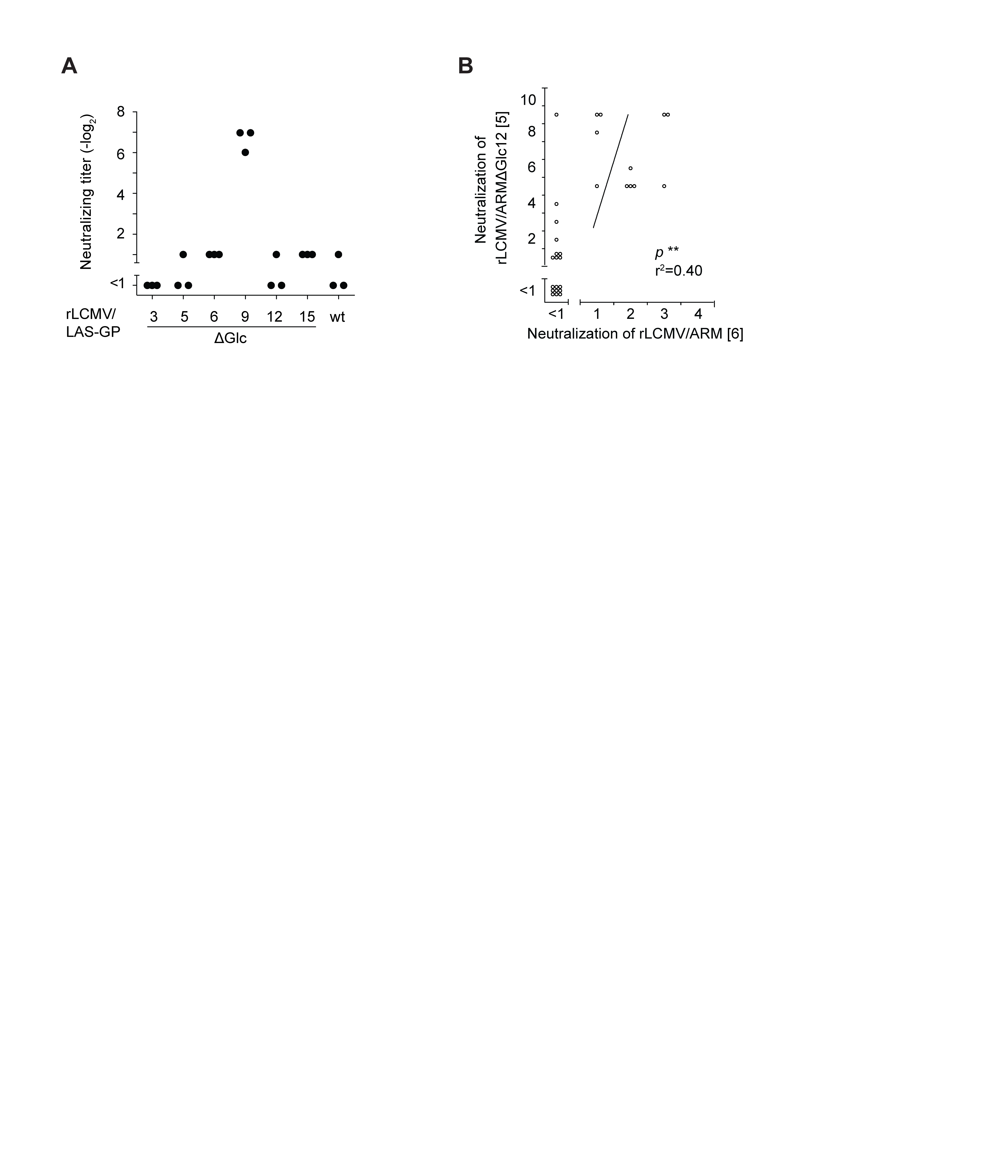

Supplement: S3 Fig — (A) Mice were primed and boosted i.v. with 104 PFU of rLCMV/LAS (wt GP) on day 0 and 59. Serum was collected on day 105 and tested in neutralization assays against rLCMV/LAS-GP variants lacking either the glycosylation motifs 3, 5, 6, 9, 12 or 15. Neutralizing titers were determined in 8-fold pre-diluted serum. Of note, we failed to recover rLCMV/LAS-GPΔGlc9, which matches analogous observations with LCMV-GP mutant viruses [35], thus corroborating a supposed structural key role of the highly conserved Glc4 in arenavirus GPs (compare Table 1). (B) We infected C57BL/6 mice with 4x10^6^ PFU of rLCMV/ARM i.v. and collected serum samples in the time window between day 60 to 67. We assessed their neutralizing activity against the immunizing rLCMV/ARM and its partially deglycosylated variant rLCMV/ARMΔGlc12, respectively. The number of N-linked glycosylation motifs in GP-1 of each variant is indicated in brackets. Each data point represents a serum sample from an individual mouse. Combined data from 29 mice in five different experiments are shown, demonstrating a positive correlation between rLCMV/ARM- and rLCMV/ARMΔGlc12-neutralizing activity. The Pearson’s correlation coefficient and two-tailed p-value are indicated. ** p<0.01. (TIF) [file ppat.1005276.s003.tif]

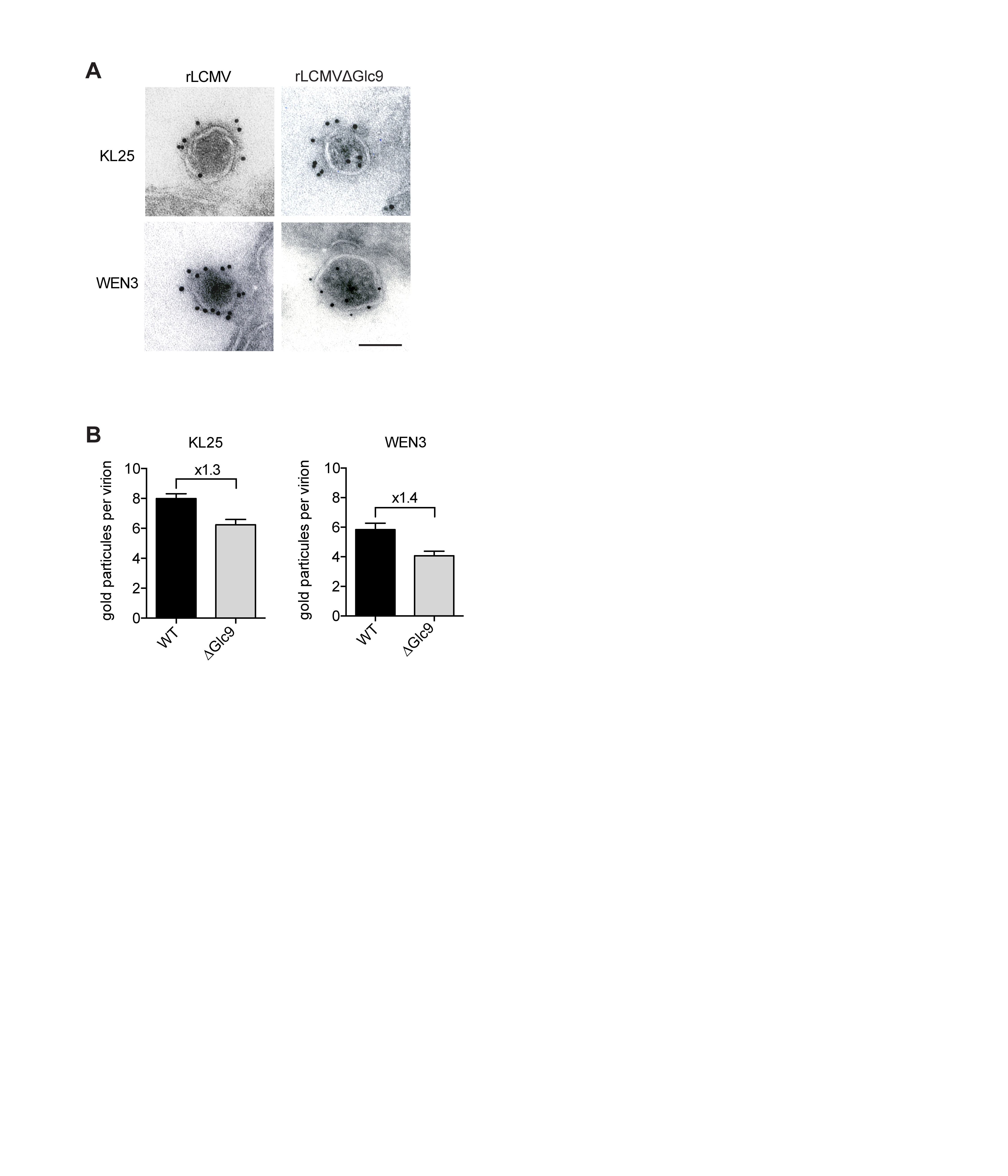

Supplement: S4 Fig — We infected BHK-21 cells with either rLCMV WT or rLCMVΔGlc9 for 48 hours and determined GP incorporation density by pre-embedding electron microscopy on budding virions. Bound KL25 or WEN3 mAbs were detected using a gold-coupled secondary antibody. (A) Representative electron micrographs showing budding virions with immunogold-labeled GP. Scale bar: 100 nm. (B) For both, KL25 and WEN3, the numbers of gold particles per virion were counted. Bars represent the mean +SEM of the following numbers of virions assessed in each staining combination. KL25 on rLCMV WT n = 204, KL25 on rLCMVΔGlc9 n = 129, WEN3 on rLCMV WT n = 88, WEN3 on rLCMVΔGlc9 n = 71. (TIF) [file ppat.1005276.s004.tif]

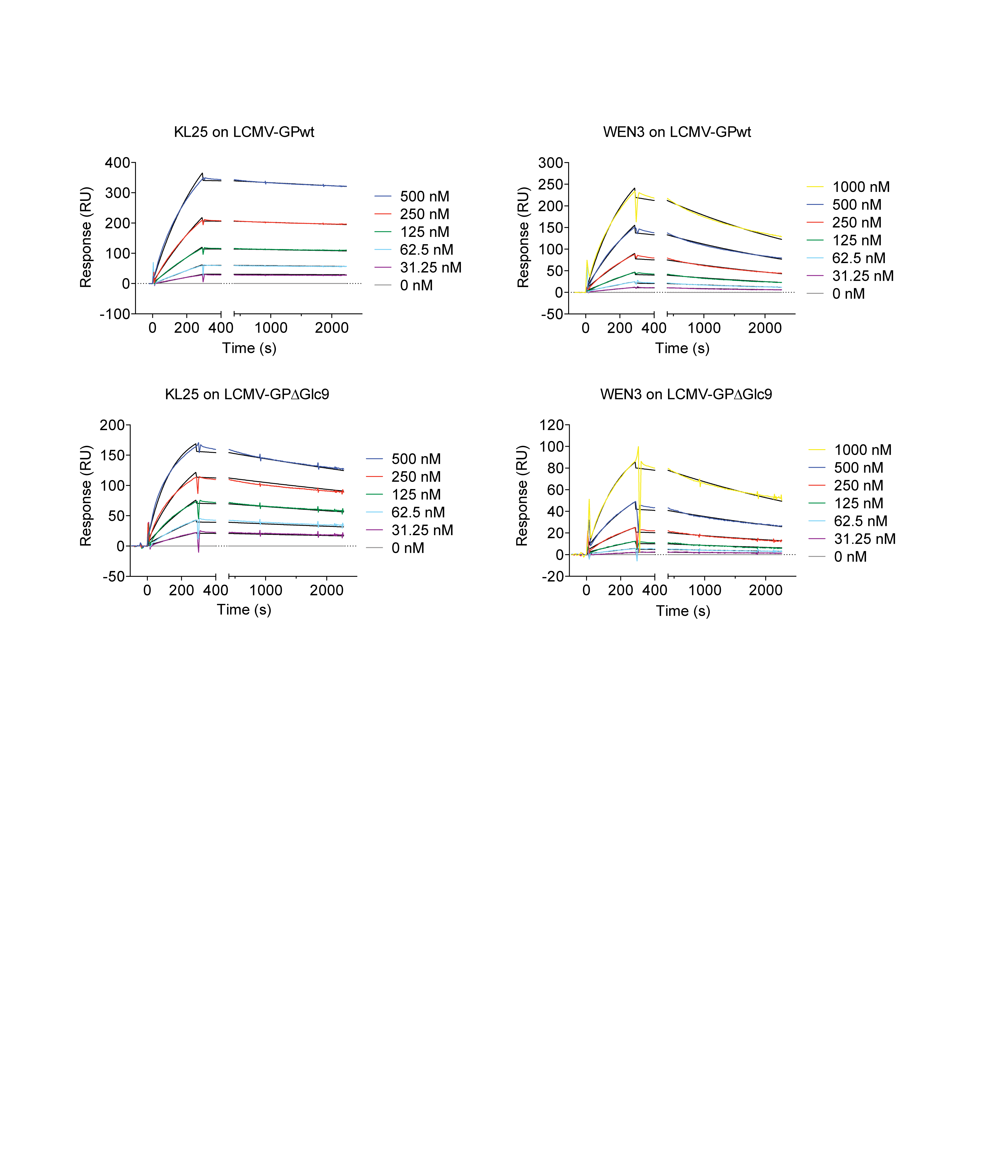

Supplement: S5 Fig — Binding kinetics of KL25 and WEN3 Fabs on LCMV-GPwt and ΔGlc9 as determined by surface plasmon resonance (SPR). The binding curves (colored lines) were globally fitted to a 1:1 Langmuir binding model (black line). Fabs were used at titrated concentrations as indicated on the graphs. For each condition, one representative binding curve out of three to four replicate measurements is shown. The corresponding binding constants are plotted in Fig 5. (TIF) [file ppat.1005276.s005.tif]

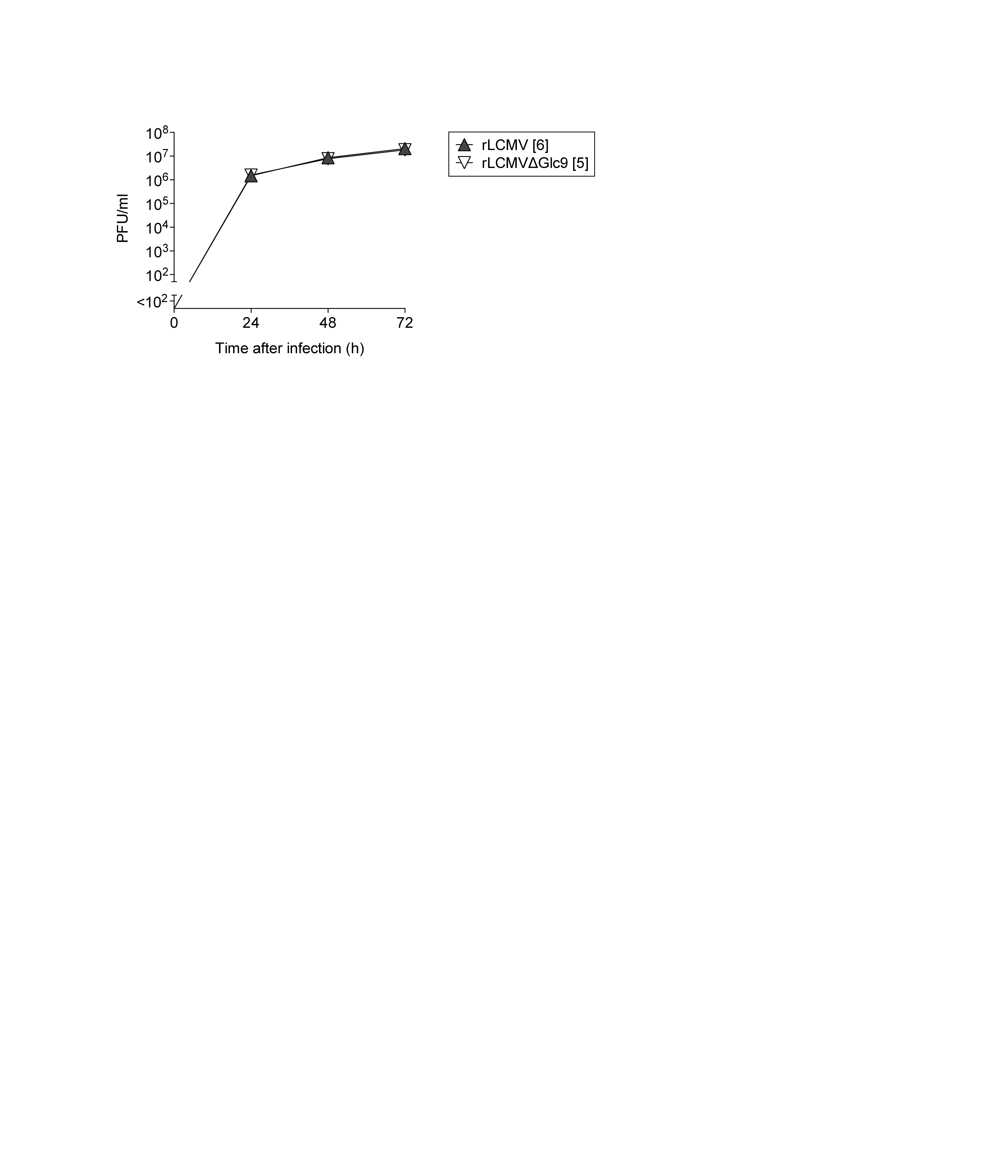

Supplement: S6 Fig — BHK-21 cells (5x10^5^ per M6 well) were infected with the indicated viruses at a multiplicity of infection of 0.01 and infectious virus in the supernatant was measured at the indicated time points. Symbols indicate the mean ±SD of three tissue culture wells (error bars project into the symbols). (TIF) [file ppat.1005276.s006.tif]
